# Cost function for low-dimensional manifold topology assessment

**DOI:** 10.1038/s41598-022-18655-1

**Published:** 2022-08-25

**Authors:** Kamila Zdybał, Elizabeth Armstrong, James C. Sutherland, Alessandro Parente

**Affiliations:** 1 Université Libre de Bruxelles, École polytechnique de Bruxelles, Aero-Thermo-Mechanics Laboratory, Brussels, Belgium; 2 Université Libre de Bruxelles and Vrije Universiteit Brussel, Combustion and Robust Optimization Group (BURN), Brussels, Belgium; 3 Department of Chemical Engineering, University of Utah, Salt Lake City, UT USA

**Keywords:** Scientific data, Computational science

## Abstract

In reduced-order modeling, complex systems that exhibit high state-space dimensionality are described and evolved using a small number of parameters. These parameters can be obtained in a data-driven way, where a high-dimensional dataset is projected onto a lower-dimensional basis. A complex system is then restricted to states on a low-dimensional manifold where it can be efficiently modeled. While this approach brings computational benefits, obtaining a good quality of the manifold topology becomes a crucial aspect when models, such as nonlinear regression, are built on top of the manifold. Here, we present a quantitative metric for characterizing manifold topologies. Our metric pays attention to non-uniqueness and spatial gradients in physical quantities of interest, and can be applied to manifolds of arbitrary dimensionality. Using the metric as a cost function in optimization algorithms, we show that optimized low-dimensional projections can be found. We delineate a few applications of the cost function to datasets representing argon plasma, reacting flows and atmospheric pollutant dispersion. We demonstrate how the cost function can assess various dimensionality reduction and manifold learning techniques as well as data preprocessing strategies in their capacity to yield quality low-dimensional projections. We show that improved manifold topologies can facilitate building nonlinear regression models.

## Introduction

In the era of big data, numerous science and engineering disciplines use dimensionality reduction to obtain lower-dimensional representations of complex physical systems with many degrees of freedom^1–8^. Large data coming from system measurements or simulations is frequently the starting point of reduced-order modeling. These high-dimensional datasets, ubiquitous in areas such as plasma physics, chemically reacting flows, neuroscience, genomics and transcriptomics, electrochemistry or atmospheric physics, often exhibit strongly attracting low-dimensional manifolds^9–20^. Describing the system evolution on those manifolds alone can thus be a viable modeling strategy^21–24^. After projecting the original variables onto a lower-dimensional basis, system dynamics can be tracked on a lower-dimensional manifold, embedded in the original state-space. This approach provides substantial reduction to the number of parameters needed to visualize, describe and predict complex systems. To date, linear and nonlinear dimensionality reduction techniques have been used to find lower-dimensional spaces to represent multivariate datasets and build reduced-order models in those spaces.

Some topological properties of low-dimensional data representations can make reduced-order modeling difficult. A particularly undesired behavior are overlapping states on a manifold which can result in non-uniqueness in dependent variable values. For instance, with the manifold parameters used as regressors in nonlinear regression tasks, any ambiguity in dependent variable values can hinder successful modeling. Another characteristic of a problematic manifold are large gradients in the dependent variable values. These can appear when observations on manifolds in important regions are compressed with respect to other, less important regions. If dependent variable values change rapidly over such compressed regions, features of small sizes are formed that can pose modeling difficulty^25^. Maintaining moderate gradients on manifolds is thus a desired characteristic.

In this paper, we are motivated by the emerging discussion on the need to characterize the quality of low-dimensional manifolds^26–30^. Quantitative tools are needed in areas where researchers tune the hyper-parameters of dimensionality reduction or manifold learning techniques to obtain improved manifolds of particular types of data^31–35^. When manifold learning is used for efficient data visualization, good quality manifolds can help uncover important multivariate relationships in complex datasets such as genomics or transcriptomics data^34,36–38^. In artificial intelligence, there is a need to determine the quality of manifold representations in neural networks, where the recent work on predictive learning demonstrated how those representations can vary throughout the learning stages^19^. Detecting intersection between several manifolds can be of interest in learning object manifolds in deep neural networks (DNNs)^39^. Similar need applies in domains that study real neural networks and aim at discovering low-dimensional trajectories in neural activity^15,18,20,40–42^. Finally, quality manifold topologies become important in fields such as fluid dynamics, plasma physics or combustion, where nonlinear regression is commonly integrated into the reduced-order modeling workflow^43–48^.

Here, we propose a manifold-informed metric that can quantitatively assess low-dimensional data parameterizations. The proposed metric takes into account two factors that affect modeling in particular: feature sizes and multiple scales of variation in manifold topology that can convey non-uniqueness. Our metric is scalable with target manifold dimensionality and can work across different dimensionality reduction and manifold learning techniques. Moreover, dependent variables that are most relevant in modeling can be selected a priori and the manifold topology can be assessed for those variables specifically.

The proposed metric can further serve as a cost function in manifold optimization tasks. In this work, we delineate and test a few applications. Primarily, we demonstrate how the cost function can help identify appropriate data preprocessing to mitigate problems with ill-behaved manifolds. While in some cases insights are available as to which data preprocessing results in accurate modeling^49–54^, such decisions still have to be largely made through trial and error. Finally, with the many linear and nonlinear dimensionality reduction techniques available in the research community, we show how they can be assessed in their capacity to generate well-defined manifolds. Our quantitative measure can optimize hyper-parameters of a reduction technique. This can be helpful especially for techniques where only heuristic guidelines are available^55^. Often, data preprocessing and hyper-parameter settings are ultimately tied to a given dataset. It is also unclear how (or whether) the settings will carry over if changes such as increasing the manifold dimensionality or extending the state-space with additional data are made later on^56^. Our approach proposed in this work thus provides a way to quantify and automate decisions that need to be made prior to applying a reduction technique.

**Figure 1:**
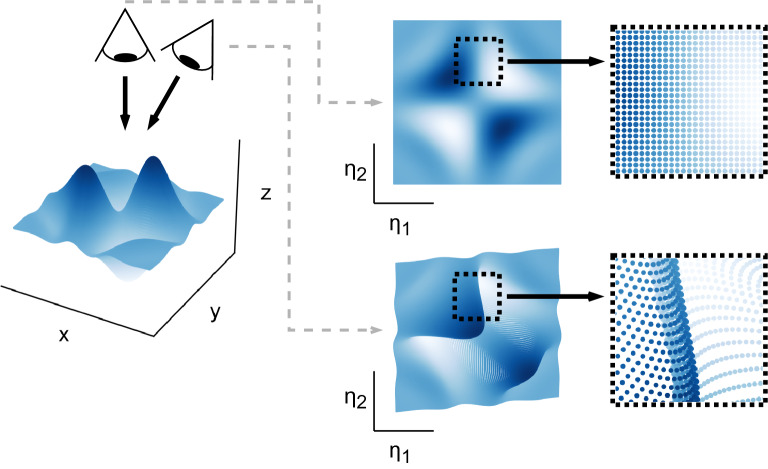
Illustrative demonstration of non-uniqueness introduced during low-dimensional data projection. Various 2D projections of a 3D synthetic dataset can be formed by looking at the dataset at various angles to the *z*-axis and collapsing all observations onto a plane of sight. We demonstrate two example projections that can be formed: a top-down projection and a projection resulting from looking at an angle to the *z*-axis. In the new projected coordinates, $[\eta _1, \eta _2]$, the top-down projection is unique everywhere, while the projection at an angle introduces regions of non-uniqueness with overlapping observations.

**Figure 2:**
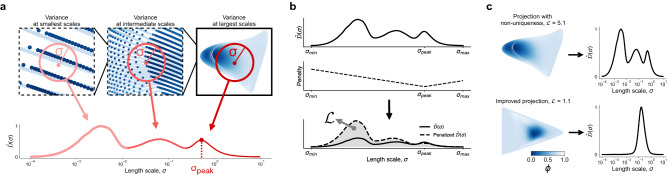
The proposed cost function, $\mathcal {L}$, distills information about variance in dependent variable’s values occurring at different length scales, $\sigma$, on a manifold. (**a**) Manifold is first scanned at different values of $\sigma$ for any variation in the dependent variable’s values. Multiple scales of variation manifest themselves in multiple peaks seen in the constructed normalized variance derivative curve, $\hat{\mathcal {D}}(\sigma )$. The location of the rightmost peak, $\sigma _{peak}$, denotes the largest feature size on a manifold. Variance occurring at scales $\sigma \ll \sigma _{peak}$ is a strong indicator of non-uniqueness. (**b**) The cost function, $\mathcal {L}$, is computed by integrating the penalized $\hat{\mathcal {D}}(\sigma )$ curve. The introduced penalty amplifies the area under $\hat{\mathcal {D}}(\sigma )$ occurring at $\sigma < \sigma _{peak}$ and especially at $\sigma \ll \sigma _{peak}$. The contributions from all scales of variation are thus summed up and embedded in the cost value. (**c**) Assessing example 2D projections with the proposed cost function. The first projection exhibits non-uniqueness, with regions where smallest and highest values of the dependent variable, $\phi$, overlap each other. The non-uniqueness is significantly reduced on the improved projection and the corresponding $\hat{\mathcal {D}}(\sigma )$ curve exhibits a single rise indicating a single feature size over the whole manifold. We show the associated costs for the two projections, with the cost for the projection with non-uniqueness being greater.

## Results

### Cost function formulation

We base our discussion on the observation that regions of poor manifold topology (such as non-uniqueness) will only affect a dependent variable if there is variation in the variable’s values over those regions (see Fig. 1). With this premise, our cost function is derived from variance in dependent variable values happening across different length scales on a manifold. As recently proposed in Ref.^30^, we compute the normalized variance of a dependent variable, $\mathcal {N}(\sigma )$, for selected length scales, $\sigma$, on a manifold. We then analyze the derivative of $\mathcal {N}(\sigma )$ with respect to $\sigma$, denoted as $\hat{\mathcal {D}}(\sigma )$. This derivative captures the information about how the normalized variance changes as the manifold is scanned at varying length scales given by $\sigma \in \langle \sigma _{min}, \sigma _{max} \rangle$. The detailed mathematical description of $\mathcal {N}(\sigma )$ and $\hat{\mathcal {D}}(\sigma )$ can be found in the “Methods” section and in Ref.^30^. Given *q* manifold parameters, $\pmb {\eta } = [\eta _1, \eta _2, \dots , \eta _q]$, the resulting normalized variance derivative, $\hat{\mathcal {D}}(\sigma )$, can be computed for each dependent variable in a relevant set, $\pmb {\phi } = [\phi _1, \phi _2, \dots , \phi _m]$. Figure 2a shows a visual example of how the normalized variance derivative assesses information content at various manifold length scales for one dependent variable, $\phi$. The length scales at which peaks occur in a variable’s $\hat{\mathcal {D}}(\sigma )$ profile indicate feature sizes. Each scale at which a dependent variable shows variation over the manifold, including variation due to non-uniqueness, will create its own imprint in $\hat{\mathcal {D}}(\sigma )$. The largest spatial scale at which the dependent variable exhibits variation (the largest feature size) on a manifold is reflected in the rightmost peak location in the $\hat{\mathcal {D}}(\sigma )$ profile, and we define it as $\sigma _{peak}$ (see Fig. 2a). In the “Methods” section, we present a more detailed discussion of how $\sigma _{peak}$ is obtained. Any smaller feature sizes appear as additional peaks for $\sigma < \sigma _{peak}$. The 2D projection seen in Fig. 2a exhibits severe non-uniqueness which manifests itself in variance occurring at very small scales, $\sigma \ll \sigma _{peak}$.

Two particularly appealing characteristics of the normalized variance derivative are taken into account for the design of our cost function. First, the locations of peaks in the $\hat{\mathcal {D}}(\sigma )$ curve convey feature sizes on manifolds. With an appropriate penalty with respect to the largest feature size, $\sigma _{peak}$, we favor manifolds that maintain large feature sizes, as those should facilitate modeling. Second, with multiple scales of variation present on manifolds, the area under the $\hat{\mathcal {D}}(\sigma )$ curve will increase due to the additional peak(s) for $\sigma < \sigma _{peak}$. The cost function proposed herein, $\mathcal {L} = \mathcal {L}(\pmb {\eta }, \pmb {\phi })$, is computed from a penalized area under the $\hat{\mathcal {D}}(\sigma )$ curve(s). By integrating the penalized $\hat{\mathcal {D}}(\sigma )$ curve(s), we sum over the effects that multiple scales of variation have on the $\hat{\mathcal {D}}(\sigma )$ profile. A visualization of the penalty function and its effect on the $\hat{\mathcal {D}}(\sigma )$ curve for the manifold introduced in Fig. 2a is shown in Fig. 2b. We see how the area under $\hat{\mathcal {D}}(\sigma )$ weighted by the penalty function is amplified at scales far from $\sigma _{peak}$, therefore penalizing the variance occurring at smaller scales more heavily. Furthermore, the cost function gives a single number representing the parameterization “cost” for a given manifold. A larger cost indicates a worse manifold topology and a lower cost indicates an improved manifold topology in terms of modeling. If the cost is computed for *m* dependent variables, a norm over all $\mathcal {L}_i$ costs can be taken to yield a single cost value for the entire manifold: $\mathcal {L} = || \mathcal {L}_i ||$, $\forall {i \in [ 1, 2, \dots , m ]}$. Figure 2c demonstrates costs computed for a single dependent variable, $\phi$, over two different 2D projections. The first projection is the same as analyzed in Fig. 2a. The second projection has an improved topology and non-uniqueness is significantly reduced. The corresponding $\hat{\mathcal {D}}(\sigma )$ curve for the improved projection exhibits a single dominant peak which indicates a single scale of variation in $\phi$ values over the entire manifold. We report the cost, $\mathcal {L} = \mathcal {L}_{\phi }$, for each projection, with the cost for the projection with non-uniqueness being greater. The mathematical description of the proposed cost function and additional details are provided in the “Methods” section.

There are three key advantages of our proposed cost function. First, manifolds obtained using any technique can be assessed. This includes any ad hoc selected manifold parameters or empirical manifolds obtained directly from training data using dimensionality reduction or manifold learning techniques. Second, manifolds of any dimensionality can be assessed. Finally, manifolds can be assessed with respect to an arbitrary set of *m* relevant dependent variables, $\pmb {\phi }$. Dependent variables that we are most interested in accurately modeling can be selected, which can include any of the original state variables and any functions of them.

**Figure 3:**
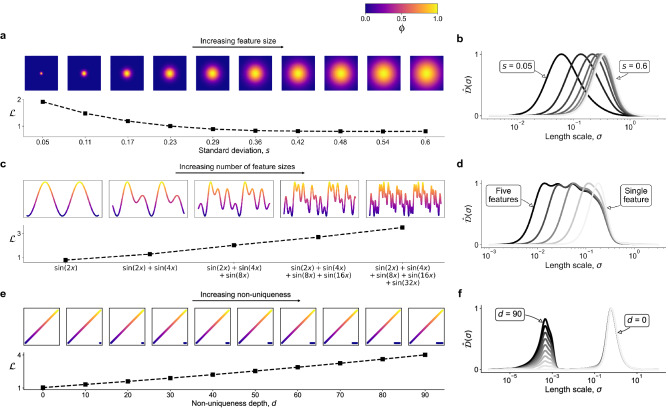
Cost function behavior on toy functions. (**a**) The response of the cost function to increasing feature size. The dependent variable $\phi$ is generated from the bivariate Gaussian normal distribution with varying standard deviation, *s*. (**b**) The $\hat{\mathcal {D}}(\sigma )$ curves corresponding to functions with increasing feature size. The curves are plotted in gray-scale denoting an increasing value of *s* from the darkest curve ($s = 0.05$) to the lightest ($s = 0.6$). (**c**) The response of the cost function to multiple feature sizes. The dependent variable $\phi$ is generated from a superposition of sine functions with varying frequencies. (**d**) The $\hat{\mathcal {D}}(\sigma )$ curves corresponding to functions with increasing number of feature sizes. The curves are plotted in gray-scale, such that the darkest curve corresponds to five different feature sizes and the lightest curve corresponds to a single feature size. (**e**) The response of the cost function to increasing non-uniqueness. The dependent variable $\phi$ is generated as a linear function of *x* with an additional set of overlapping observations for which $\phi = 0$. (**f**) The $\hat{\mathcal {D}}(\sigma )$ curves corresponding to functions with increasing non-uniqueness. The curves are plotted in gray-scale denoting an increasing value of the non-uniqueness depth, *d*, from the lightest curve ($d=0$) to the darkest ($d=90$). In all examples, the dependent variables, $\phi$, have been normalized to a [0, 1] range.

### Cost function response to feature size and non-uniqueness

To demonstrate the behavior of the proposed cost function to an increasing feature size, we generate bivariate functions on a uniform square *x*–*y* grid, centered around (0, 0). The dependent variable, $\phi$, is calculated from a multivariate Gaussian normal distribution, $\phi = \text {exp} ( - (x^2 + y^2) / (2 s^2))$, where the standard deviation, *s*, is gradually increased to imitate an increasing feature size. The smallest feature is selected with $s = 0.05$ and the largest feature with $s = 0.6$. Figure 3a shows these functions corresponding to ten Gaussians with increasing *s*. Below each Gaussian, we plot the corresponding cost, $\mathcal {L} = \mathcal {L}_{\phi }$, and we observe a decreasing trend in $\mathcal {L}$ with increasing feature size. This is a desired behavior, since larger features should facilitate modeling. A very small feature, like the one obtained from $s=0.05$, introduces relatively steep gradients, which can be more challenging to model. The response of the $\hat{\mathcal {D}}(\sigma )$ curves to an increasing feature size is shown in Fig. 3b. The location of $\sigma _{peak}$ gradually shifts to the right with increasing feature size and the area under the $\hat{\mathcal {D}}(\sigma )$ curves decreases. The decrease in cost with an increasing feature size seen in Fig. 3a is thus a result of rewarding the increasing $\sigma _{peak}$ location and the slightly decreasing area under the $\hat{\mathcal {D}}(\sigma )$ curve. The latter is the effect of a decreasing gradient of $\phi$ with increasing feature size, leaving less variance in $\phi$ present at scales away from $\sigma _{peak}$.

To further test the response of the cost function to multiple feature sizes, we generate another set of functions, such that the dependent variable, $\phi$, is now computed from a superposition of sine functions with various frequencies. Multiple feature sizes are generated from the general formula $\phi = \sum _{k=1}^{n} \sin (2^{k} x)$ for $n = 1, 2, \dots , 5$. Thus, for $n=1$, the function has only one feature size. In Fig. 3c, we observe increasing cost values with each added feature size. The reason for this increase becomes clear once we look at the corresponding $\hat{\mathcal {D}}(\sigma )$ curves in Fig. 3d, where each new feature generates an additional rise in $\hat{\mathcal {D}}(\sigma )$ at length scales smaller than $\sigma _{peak}$.

Next, we perform a similar test using functions which introduce increasing levels of non-uniqueness. The dependent variable, $\phi$, is now calculated as a linear function of the independent variable, $\phi = x$, being the *x*-axis. This results in a constant gradient along the *x*-axis. We introduce non-uniqueness in $\phi$ by adding overlapping observations whose $\phi$ values are set to zero. The number of observations added to increase the non-uniqueness is measured with the overlap depth, *d*, and is increased from no overlapping observations ($d=0$ observations) to a maximum number of overlapping observations ($d=90$ observations). Figure 3e presents ten such functions with an increasing depth of non-uniqueness. Here, we observe an increasing trend in $\mathcal {L}$ as we increase the non-uniqueness depth. The multiple scales of variation introduced from non-uniqueness lead to an increased area under the $\hat{\mathcal {D}}(\sigma )$ curves, which is penalized by the cost function. In order to observe how severe the increase in area is under the $\hat{\mathcal {D}}(\sigma )$ curves associated with manifold non-uniqueness, Fig. 3f shows the $\hat{\mathcal {D}}(\sigma )$ curves corresponding to the ten functions with non-uniqueness. The first interesting observation is that the location of $\sigma _{peak}$ for all $\hat{\mathcal {D}}(\sigma )$ curves is almost identical. This is understandable, since the size of the main feature (the constant gradient) stays the same. The only reason for the increased area under the $\hat{\mathcal {D}}(\sigma )$ curves is the appearance of additional peaks at length scales $\sigma \ll \sigma _{peak}$. Only in the case $d=0$ (no overlap) the $\hat{\mathcal {D}}(\sigma )$ curve exhibits a single rise. As long as any overlapping observations are introduced, an additional area shows up under the $\hat{\mathcal {D}}(\sigma )$ curve at $\sigma \ll \sigma _{peak}$. This additional area is most pronounced for the largest non-uniqueness depth, $d=90$.

Finally, it is instructive to discuss the scales, $\sigma$, at which we detect overlap in Fig. 3f. The highest rise in $\hat{\mathcal {D}}(\sigma )$ linked to non-uniqueness is happening at $\sigma \approx 5 \times 10^{-4}$ for all cases $d>0$. This exact value can be linked to the sample density for the ten toy functions with increasing overlap depth. For a normalized manifold, where $x \in [0,1]$, the distance along the *x*-axis between data points in the unique region is $\sigma \approx 10^{-3}$. The overlapping observations are located in-between every other unique observation, such that the distance along the *x*-axis between any observation from the unique region and its nearest overlapping observation is of the order of $10^{-4}$. Thus, as the manifold is scanned with varying length scales, $\sigma$, once $\sigma \approx 10^{-4}$, the variation in a dependent variable values is captured between a single point from the unique region ($\phi \rightarrow 1$) and a single point from the overlapping region ($\phi =0$). This creates a sudden increase in the captured variance and shows up as the peak at $\sigma \approx 5 \cdot 10^{-4}$ in the $\hat{\mathcal {D}}(\sigma )$ curves in Fig. 3f. A more in-depth discussion of how scales of variation can be linked to data density on a manifold grid can be found in Ref.^30^.

**Figure 4:**
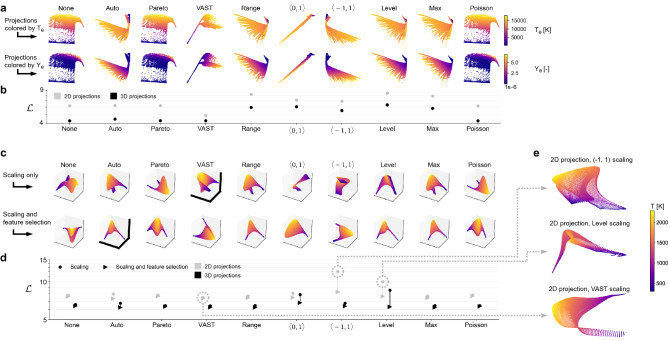
Ranking of different training data preprocessing strategies in their capacity to produce quality low-dimensional projections using PCA. (**a**) 2D PCA projections generated from an argon plasma dataset. We show projections resulting from various scaling applied to the original data. In the top row, the projections are colored by the electron temperature, $T_e$, and in the bottom row, by the electron mass fraction, $Y_e$. (**b**) The overall cost, $\mathcal {L}$, computed as the L$_1$-norm over the individual costs for the selected dependent variables: temperature of heavy species, $T_h$, temperature of electrons, $T_e$, electron mass fraction, $Y_e$, and argon mass fraction, $Y_{Ar}$. For comparison, costs are shown for 2D and 3D PCA projections. (**c**) 3D PCA projections generated from a reacting flow dataset describing combustion of syngas in air. We compare projections resulting from only scaling the data (top row) with projections resulting from scaling combined with feature selection (bottom row). All projections are colored by the temperature variable. (**d**) The overall cost, $\mathcal {L}$, computed as the L$_1$-norm over the individual costs for the selected dependent variables: temperature and six important chemical species mass fractions. We compare costs corresponding to only scaling the data (circles) versus scaling with feature selection (triangles). We also compare costs corresponding to the 3D projections visualized above (black markers) with the analogous costs of 2D projections (gray markers). The optimal manifold topology corresponding to the lowest $\mathcal {L}$ for each preprocessing strategy is highlighted with thicker axes in (**c**). (**e**) Visualization of 2D projections corresponding to three selected cases of only scaling the original data: $\langle -1, 1 \rangle$ and Level scaling (corresponding to the two highest $\mathcal {L}$) and VAST scaling (corresponding to the lowest $\mathcal {L}$).

### Assessing data preprocessing strategies

We now turn our attention to multivariate datasets and their lower-dimensional projections. Each dataset considered next is represented by a matrix $\mathbf {X} \in {\mathbb {R}^{N \times Q}}$, where *N* is the number of observations and *Q* is the number of state variables. Since most often $N \gg Q$, *Q* determines data dimensionality. We consider three disciplines that deal with datasets that are notoriously high dimensional: plasma physics, reacting flows and atmospheric physics. For example, the state-space of a reacting flow is described by the temperature, pressure and chemical composition. There, the high state-space dimensionality originates from many chemical species involved that can easily reach the order of hundreds. In this and the following sections, we demonstrate a few practical applications of the proposed cost function using those datasets.

Reduced-order models leverage the fact that multivariate datasets can often be successfully re-parameterized by a reduced set of low-dimensional parameters. To date, numerous techniques have been employed for dimensionality reduction of multivariate data. Those include linear techniques such as principal component analysis (PCA), independent component analysis (ICA), distance metric learning (DML) or linear discriminant analysis (LDA) and nonlinear techniques such as kernel PCA (KPCA), Isomap^57,^locally linear embedding (LLE) and its variants^58^ or autoencoders^59^. For the purpose of data visualization, t-distributed stochastic neighbor embedding (t-SNE)^55^ and uniform manifold approximation and projection (UMAP)^60^ are gaining popularity in various research disciplines^7,36–38,61–64^. A short summary of dimensionality reduction and manifold learning techniques explored in this work can be found in the “Methods” section.

Prior to applying dimensionality reduction, training datasets are often preprocessed. The most straightforward strategy is data normalization by centering and scaling each state variable. Other preprocessing approaches involve data sampling to mitigate imbalance in observation density, or feature selection. The effect of data preprocessing alone can have a large impact on the resulting low-dimensional manifold topology constructed from such data^53,65^. To demonstrate how significant those changes can be, Fig. 4a shows 2D PCA projections of a dataset describing argon plasma (*N* = 100,700, *Q*=36)^66,67^, where the state-space is spanned by the following variables: temperature of heavy species, $T_h$, temperature of electrons, $T_e$, and 34 species mass fractions that contain 31 electronic states of argon, two levels of ionized argon, Ar and $\hbox {Ar}^{+}$, and electrons. The projections are generated with various scaling techniques applied on the dataset (see the “Methods” section for the summary of all scaling techniques used in this work). In the top row of Fig. 4a, the projections are colored by $T_e$ and in the bottom row by the electron mass fraction, $Y_{e}$. Our visual analysis shows that preprocessing can affect manifold topologies significantly. Various topologies are expected to perform differently in reduced-order modeling due to changes in feature sizes or the level of non-uniqueness.

We quantitatively assess those changes and rank the preprocessing strategies in their capacity to generate quality manifold topologies. In Fig. 4b, we show costs, $\mathcal {L}$, for the various 2D PCA projections visualized above, and for comparison, for the analogous 3D PCA projections (although not visualized). Here, $\mathcal {L}$ is computed as the L$_1$-norm over the individual costs for the relevant dependent variables: $T_h$, $T_e$, $Y_{e}$ and $Y_{Ar}$. Thus, in Fig. 4a, the projections are colored by the two target dependent variables to allow us for making a visual connection between how the projection topologies look like versus how the cost function assesses a given projection. We note that VAST scaling results in the lowest cost for 2D projections. Looking at the corresponding visualization in Fig. 4a, we can see that $T_e$ and $Y_{e}$ values change relatively smoothly across the manifold, even though the VAST projection introduces the “spike” region, where many data observations are compressed. For comparison, projections resulting from the remaining scalings introduce relatively steep gradients and overlap for a few of them, in some region of the projection, either for the $T_e$ or the $Y_{e}$ variable. Finally, we note that costs drop across all scaling strategies explored when the PCA projection dimensionality is increased to 3D.

We additionally explore feature selection (otherwise known as variable subset selection) as another strategy in the data preprocessing pipeline. Feature selection involves finding a meaningful subset, $\mathbf {X}_S \in {\mathbb {R}^{N \times S}}$, of the original *Q* state variables ($S < Q$), and using this subset in data science or machine learning algorithms, instead of the full set of state variables^68^. This time, we use a reacting flow dataset describing combustion of syngas in air. In Fig. 4c, we show 3D PCA projections of an 11-dimensional dataset (*N* = 14,550, *Q* = 11), where the eleven dimensions are spanned by temperature, *T*, and ten mass fractions of chemical species, denoted $Y_i$ for species *i*. The different projections seen in Fig. 4c correspond to different preprocessing applied to the original combustion dataset. In the upper row, the 3D projections are generated with various scaling techniques applied on the training dataset (see “Methods”). The bottom row shows 3D projections resulting from different scalings combined with feature selection applied to the original training data. All PCA projections are colored by the temperature. For the purpose of this demonstration, we use a newly developed feature selection algorithm that uses the cost function to guide the optimal selection of the variables from the original training data^65^. By minimizing the cost of the resulting data projection, we optimize the feature selection process from the point of view of manifold topology. The detailed description of the feature selection algorithm can be found in the “Methods” section. In Fig. 4d we show costs, $\mathcal {L}$, tied to the two preprocessing strategies explored: just scaling the data (circles) versus scaling with feature selection (triangles). Here, $\mathcal {L}$ is computed as the L$_1$-norm over the individual costs for the relevant dependent variables: *T*, $Y_{H_2}$, $Y_{O_2}$, $Y_{H_2O}$, $Y_{OH}$, $Y_{CO}$ and $Y_{CO_2}$. The dependent variables were selected manually as the most important candidates in modeling. Black markers show costs corresponding to the 3D PCA projections visualized in Fig. 4c. For comparison, with gray markers we show costs for analogous 2D PCA projections. Three of these 2D projections corresponding to scaling only are visualized in Fig. 4e—the two worst projections (largest $\mathcal {L}$) and the best projection (smallest $\mathcal {L}$). We note that the worst 2D projection corresponding to $\langle -1, 1 \rangle$ scaling is severely folded over itself which is likely the reason for the high cost. The Level projection also introduces significant non-uniqueness, with the low-temperature regions being represented over narrow geometry on a manifold. The best 2D projection corresponds to VAST scaling. As seen in Fig. 4e, this projection is much more unique compared to $\langle -1, 1 \rangle$ or Level scaling 2D projections. However, there is a visible “twist” on the VAST 2D projection, which is untangled when a 3D projection is considered instead. This is a possible factor for a decreased cost between the 2D and the 3D PCA projection. The lowest costs for 3D projections happened for VAST (for scaling only) and Auto (for scaling with feature selection). The corresponding visualized projections are marked with thicker axes in Fig. 4c. These two projections are characterized by relatively well-spaced feature sizes and reduced non-uniqueness. Finally, similarly as we have observed for the argon plasma data, costs drop across all scaling strategies when the PCA projection dimensionality is increased from 2D to 3D.

Our analysis from Fig. 4 reveals that appropriate data preprocessing can help in generating a better quality low-dimensional manifold. In particular, optimized feature selection can be beneficial as it decreases costs over the relevant dependent variables with respect to costs associated with only scaling the data. With the many possible data preprocessing techniques encountered in the data science community, the cost function allows for quantitative rankings without the need to analyze manifold topologies manually. This, in turn, can help fine-tune data preprocessing to a given dataset and a desired manifold dimensionality.

### Detecting large gradients on manifolds

**Figure 5:**
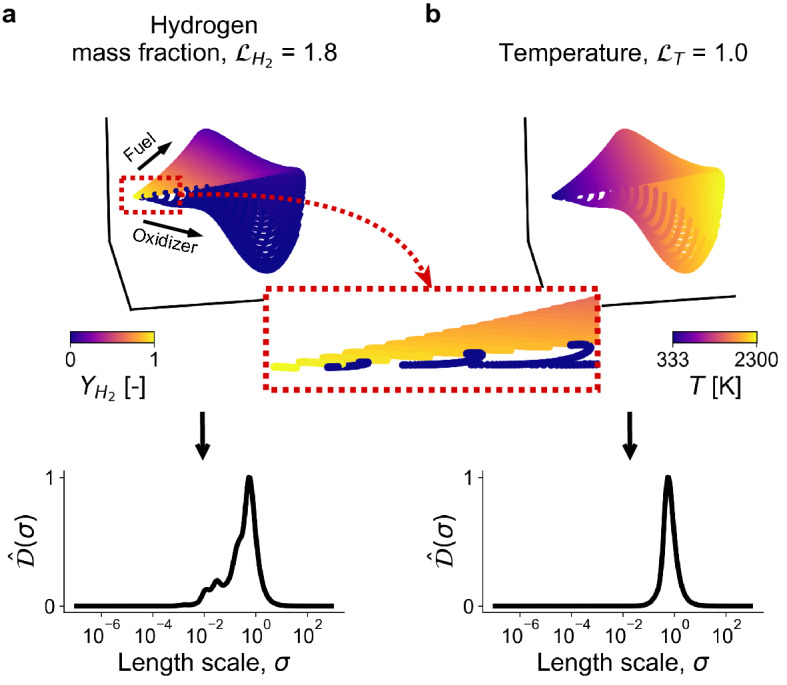
Example 3D manifold in a reacting flow dataset describing combustion of hydrogen in air. (**a**) The manifold exhibits region (marked with the red dashed box) with a step-change in hydrogen (fuel) mass fraction values, $Y_{H_2}$. (**b**) For comparison, the temperature variable, *T*, on the same manifold does not experience large variation within the considered region and the cost associated with the temperature variable is lower.

Using the proposed cost function, manifolds can be assessed with respect to different relevant dependent variables separately. If there is a good coverage of regions where the selected variables vary over the entire manifold, problematic regions on manifolds can be detected with greater reliability. Figure 5a demonstrates a 3D manifold embedded in a 9-dimensional state-space of a reacting flow dataset describing combustion of hydrogen in air (*N* = 13,468, *Q* = 9). The topology of this manifold is curved such that two regions where fuel and oxidizer originate from are brought closely together. This region is better visualized in the zoomed-in dashed box. When the manifold is colored by the hydrogen (fuel) mass fraction in Fig. 5a, we see a step-change in the $\phi = Y_{H_2}$ values over the considered region. This potentially undesired behavior can be detected with the cost function as it increases the area under the $\hat{\mathcal {D}}(\sigma )$ curve at length scales smaller than $\sigma _{peak}$. The cost associated with the hydrogen mass fraction is $\mathcal {L}_{H_{2}} = 1.8$. For comparison, the temperature variable does not experience large variation within the considered region (Fig. 5b) and the cost associated with temperature is lower ($\mathcal {L}_T = 1.0$). We observe a single peak in the $\hat{\mathcal {D}}(\sigma )$ profile corresponding to the temperature variable.

Regions where opposing physical phenomena meet over small length scales on a manifold (in this case fuel and oxidizer streams) can prove to be difficult to model accurately. For instance, some regression models are known to struggle in the presence of large gradients in dependent variable values^25^. Such problematic regions of compressed observations on manifolds can be detected by analyzing costs across several dependent variables. If there exists an important dependent variable that varies across the problematic region (such as the $Y_{H_2}$ variable in Fig. 5a), it can help discard the problematic manifold topology. We note that the choice of the relevant dependent variables is important in assessing whether a given manifold topology is appropriate or not from the modeling perspective. Some variables, such as the temperature variable alone in the example shown in Fig. 5b, might not be effective at exposing regions of non-uniqueness in data projections, that can affect other relevant variables. We also note that the dependent variables for which the cost is computed can be arbitrary and do not have to be selected from the set of the original state variables as we have done here. They can equally be functions of the state variables. In reacting flow applications, these can for instance be the production rates of chemical species, related to the state-space through nonlinear Arrhenius expressions.

### Manifold assessment across dimensionality

**Figure 6:**
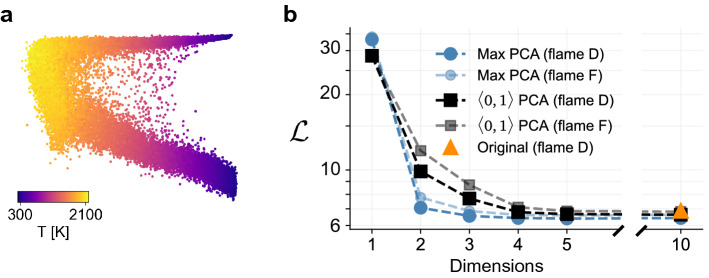
Cost function applied to PCA projections of experimental Sandia flame data. (**a**) A 2D projection of flame D using PCA with Max scaling colored by temperature. (**b**) The overall cost, $\mathcal {L}$, computed as the L$_1$-norm over the individual costs for selected dependent variables: temperature and six important chemical species mass fractions when using PCA for reduction between one and ten dimensions. The figure includes curves for using Max and $\langle 0, 1 \rangle$ scalings with PCA as well as the cost for the original ten-dimensional manifold.

We now demonstrate how the cost function applies to manifolds of arbitrary dimensionality. This can be useful in determining an appropriate dimensionality for representing the variables of interest when using a reduction technique. The demonstration is first done using an experimental combustion dataset known as the Sandia flame D dataset^69^. This dataset contains approximately *N* = 57,000 observations each for temperature and various species mass fractions (*Q* = 10) over six different heights in a methane and air piloted jet flame. The experimental dataset also presents the opportunity to demonstrate the cost function behavior on manifolds containing noise. Figure 6a shows an example 2D PCA projection with Max scaling of the Sandia flame D data colored by temperature. This manifold is less structured than those seen in Figs. 4 and 5 for the numerically simulated data. With noisy data, such as seen from experiment, it can be difficult to visually assess the quality of manifold topologies, especially in higher dimensions. The proposed cost function should adequately assess such topologies since the $\hat{\mathcal {D}}(\sigma )$ curves remain smooth even for noisy data^30^.

Figure 6b demonstrates how the cost function behaves with increasing dimensionality of a projection using PCA, up to the original dimensionality of the system. The two opaque curves correspond to the scalings that resulted in the highest and lowest costs for the projected experimental data. Also included is the cost for the original ten-dimensional manifold. For the most part, we see a decrease in the cost with increasing dimensionality. Increasing the dimensionality further after four dimensions appears to have negligible effects on the feature sizes of the optimizing variables since the cost function flattens out. At this point, the computational cost of adding dimensions to a model most likely outweighs any benefit seen from slight increases in feature sizes. Any differences between the various scalings also become less noticeable as dimensionality increases. We also see that rotating the manifold along new principal component axes, even without reducing dimensionality, can slightly reduce the cost compared to the original manifold. This is due to variance being measured along new coordinates. For comparison, in Fig. 6b we also include costs with increasing dimensionality for the Sandia flame F dataset (transparent curves), which is another experimental case that exhibits stronger effects of flow turbulence than flame D. As a result, flame F experiences conditions with local flame extinction, absent in the flame D dataset. Thus, flame F includes many more states of methane combustion than flame D and the manifold corresponding to flame F covers a wider range of attainable thermo-chemical states. For instance, for a specific stoichiometric condition, the flame can be either in an ignited or in an extinguished state. We thus expect an increased required manifold dimensionality for flame F to achieve the same parameterization quality as compared to flame D. This is indeed the case seen in Fig. 6b, where costs are generally higher for flame F than for flame D. The proposed cost function could be used in this way to determine an appropriate minimum dimensionality for the projection of data, with the goal of facilitating modeling of the optimizing variables^70,71^.

### Manifold assessment across various dimensionality reduction and manifold learning techniques

**Figure 7:**
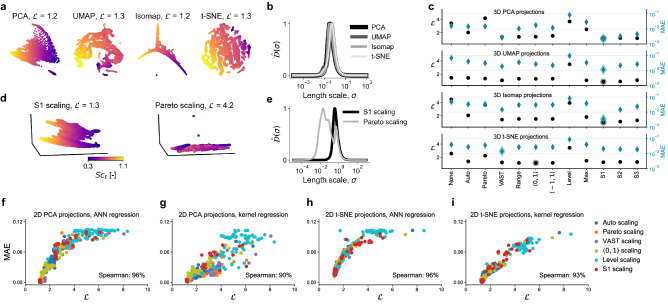
Relating the cost function to nonlinear regression predictions based on various low-dimensional projections of the atmospheric pollutant dispersion data. The relevant dependent variable is the turbulent Schmidt number, $\phi = Sc_t$. (**a**) 2D data projections using PCA, UMAP, Isomap and t-SNE with the cost for $\phi = Sc_t$ reported for each projection. (**b**) The $\hat{\mathcal {D}}(\sigma )$ curves corresponding to the 2D projections. (**c**) Ranking of different data scaling techniques using the cost function (black dots) with the mean absolute errors (MAE) for ANN prediction of $Sc_t$ across all four projection techniques (blue diamonds) using 3D data projections. The minimum $\mathcal {L}$ and the minimum MAE for each technique is marked with a shaded outline. Scalings that generally exhibit lowest costs (VAST, S1, S2, S3) also result in the smallest MAE. (**d**) Example 3D PCA projections resulting from applying two scaling options to the original data: S1 and Pareto scaling. For this dataset, S1 scaling allowed for generating a manifold where the dependent variable of interest, $Sc_t$, has a smooth gradient across one of the manifold dimensions. Pareto scaling, exhibiting high cost, collapses most of the data observations onto a planar structure. (**e**) The $\hat{\mathcal {D}}(\sigma )$ curves corresponding to the 3D PCA projections with S1 and Pareto scaling. (**f**–**i**) Scatter plots of $\mathcal {L}$ versus MAE from ANN and kernel regression predictions of $Sc_t$. We show predictions based on 600 different 2D PCA projections (**f**, **g**), and based on 600 different 2D t-SNE projections (**h**, **i**) as the independent manifold parameters. We test a few selected scaling techniques applied to the atmospheric dispersion data; legend applies to all figures (**f**–**i**).

We now revisit a recent PCA-based reduction of a high-fidelity dataset obtained from delayed detached eddy simulation in atmospheric physics^46^. This data simulates the Cedval A1–5 wind tunnel measurements of atmospheric dispersion of a pollutant in the vicinity of a rectangular building^72^. The numerical data that we use here represents a planar slice through the computational domain, perpendicular to the downstream wind direction. The dataset has *N* = 18,540, *Q* = 16 and the original state-space is composed of variables common to atmospheric physics applications, such as the three velocity components, pressure, Reynolds number, or the turbulent viscosity, kinetic energy and dissipation rate. All parameters in the data have been averaged over a 3.5 s period. We use an outlier detection algorithm to remove outliers from the data (see "Methods"). The aim of the original study^46^ was to predict the turbulent Schmidt number, $Sc_t$, from the PCA-derived manifold parameters. This demonstrates an application where regression is the end goal for obtaining a data-driven correlation of a physical quantity with the manifold parameters, $\pmb {\eta }$. We thus seek such regression function *f* that $Sc_t \approx f(\pmb {\eta })$. While in the original work PCA was used to reduce the state-space dimensionality, here we benchmark three additional techniques: UMAP, Isomap and t-SNE. Following a similar methodology as demonstrated in Fig. 4, we first searched for the best scaling option for each reduction technique. In addition, we introduce three novel scaling methods inspired by the variable stability (VAST) scaling^49^. We denote them S1, S2 and S3 (see Table 1). S1 scaling is an extension of VAST, where the effect of non-normality is considered by multiplying the standard deviation by the data kurtosis. S2 and S3 are variations of S1 obtained by replacing the mean value in the coefficient of variation by the maximum and the range of each variable respectively. In Fig. 7a, we compare qualities of 2D projections, corresponding to the best scaling, both visually and using our cost function. We report costs for the $\phi = Sc_t$ only, since that was the modeled dependent variable. An appropriate scaling allowed us, in many cases, to find significantly better projections. For instance, the worst 2D PCA projection is associated with $\mathcal {L} = 5.2$, while the best (visualized) with $\mathcal {L} = 1.2$. Figure 7b shows the $\hat{\mathcal {D}}(\sigma )$ curves corresponding to the 2D projections visualized in Fig. 7a and to $\phi = Sc_t$. The $\hat{\mathcal {D}}(\sigma )$ curves are mostly composed of a single rise, suggesting that the non-uniqueness in the 2D projections has been remedied by selecting an appropriate data scaling.

**Table 1:** Data scaling techniques used in this work.

Name	Scaling factor $d_j$	Center $c_j$
None	1	0
Auto^51^	$s_j$	$\bar{X}_j$
Pareto^52^	$\sqrt{s_j}$	$\bar{X}_j$
VAST^49^	$s_j^2 / \bar{X}_j$	$\bar{X}_j$
Range^51^	$\text {max}(X_j) - \text {min}(X_j)$	$\bar{X}_j$
$\langle 0, 1 \rangle$	$\text {max}(X_j) - \text {min}(X_j)$	$\text {min}(X_j)$
$\langle -1, 1 \rangle$	$1/2 (\text {max}(X_j) - \text {min}(X_j))$	$1/2 (\text {max}(X_j) + \text {min}(X_j))$
Level^51^	$\bar{X}_j$	$\bar{X}_j$
Max	$\text {max}(X_j)$	$\bar{X}_j$
Poisson^50^	$\sqrt{\bar{X}_j}$	$\bar{X}_j$
S1	$s_j^2 k_j^2 / \bar{X}_j$	$\bar{X}_j$
S2	$s_j^2 k_j^2 / \text {max}(X_j)$	$\bar{X}_j$
S3	$s_j^2 k_j^2 / ( \text {max}(X_j) - \text {min}(X_j))$	$\bar{X}_j$

### Improved manifold topologies yield more accurate regression

Nonlinear regression can be used in combination with dimensionality reduction to provide mapping functions, *f*, between the manifold parameters and physical quantities of interest. Regression can help discover robust relationships between manifold parameters and physical quantities that can then be injected in computational models and simulations^18,44,46,73^. Techniques, such as artificial neural networks (ANNs), Gaussian process regression (GPR) or kernel regression are commonly used in this context.

Below, we continue the example of the atmospheric dispersion dataset and demonstrate improvements in nonlinear regression performance when parameters of an improved manifold topology are used as regressors. We first train an ANN model to predict $Sc_t$ based on 3D PCA, UMAP, Isomap and t-SNE projections resulting from different data scaling options. The details on the ANN model used here are provided in the "Methods" section. The scalings ranking for these 3D projections, along with the mean absolute errors (MAE) for $Sc_t$ predictions using ANN are reported in Fig. 7c. We note that generally, the scalings which resulted in lowest costs (VAST, S1, S2 and S3) across all four reduction techniques exhibit lowest MAE. The minimum $\mathcal {L}$ and MAE for each technique is marked with a shaded outline. With the exception of t-SNE, the minimum $\mathcal {L}$ and the minimum MAE happened for the same scaling option.

The reason for a good ANN performance on those four scalings can be further understood by visualizing example projections. In Fig. 7d, we show 3D PCA projections of the atmospheric dispersion data, corresponding to the best scaling option (S1 with $\mathcal {L} = 1.3$) and the worst scaling option (Pareto with $\mathcal {L} = 4.2$) as per the ranking shown in Fig. 7c. The quality of projections changes visibly with the change of data scaling. For the S1 scaling projection, we observe a clear gradient of $Sc_t$ throughout the manifold, while for the Pareto scaling case, the 3D projection introduced significant scatter in $Sc_t$ values, with many observations compressed onto a nearly planar structure in the reduced 3D space. The latter projection thus introduces significant non-uniqueness in $Sc_t$ values and is more difficult to accurately regress over. The difference in costs for the two 3D PCA projections from Fig. 7d can be further understood by looking at the comparison of $\hat{\mathcal {D}}(\sigma )$ curves in Fig. 7e. Pareto scaling case creates much more variance present at smaller length scales which is consistent with our visual inspection of the projection.

More generally, we observe correlation between the verdict given by $\mathcal {L}$ and the nonlinear regression performance. In Fig. 7f–i, we show scatter plots of $\mathcal {L}$ versus MAE for varying manifold topologies obtained from the atmospheric dispersion dataset. Here, we focus on PCA and t-SNE as the two projection techniques. Each scatter plot takes into account six selected scaling techniques: Auto, Pareto, VAST, $\langle 0, 1 \rangle$, Level and S1. For each scaling technique, we generate 100 distinct 2D projections through creating random variable subsets (feature selections) of the full dataset, before applying PCA or t-SNE. This allows us to have sufficiently many distinct manifold topologies (600 for each scatter plot) for a trend to emerge in Fig. 7f–i. In addition to ANN regression, we apply kernel regression of $Sc_t$ to observe if the correlation between $\mathcal {L}$ and MAE is still present for a different nonlinear regression technique. The details on the kernel regression model used here are provided in the “Methods” section. Although the trends of $\mathcal {L}$ vs. MAE are different between ANN and kernel regression, the correlation measured with Spearman coefficient is high in each case. For ANN regression we observe 96% Spearman correlation for 2D PCA and t-SNE projections. The correlation is lower for kernel regression as compared to ANN regression, with 90% and 93% Spearman coefficient for PCA and t-SNE respectively. In Fig. 7f–i, manifolds corresponding to Level scaling are clustered in the region of high $\mathcal {L}$ and high MAE. This result is consistent with Fig. 7c, where Level scaling is seen to yield high costs and high regression errors. Conversely, the region of smallest $\mathcal {L}$ and smallest MAE in Fig. 7f–i is occupied by topologies resulting from S1 scaling, although some random subsets can result in poorer manifold topologies even with S1 scaling. We observe similar correlation trends for 3D PCA and t-SNE projections; these are shown in the Supplementary material. With the regression hyper-parameters kept constant throughout this exercise, the results presented in Fig. 7f–i suggest that better regression performance can be achieved when manifold topologies are improved. The cost function can thus be used to find optimized regressors for nonlinear regression techniques such as ANN or kernel regression. Other regression techniques are expected to be similarly affected.

**Figure 8:**
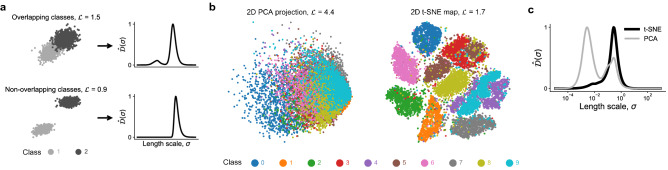
Using the cost function to detect overlap between classes in categorical data. (**a**) Toy dataset composed of two clouds. The first case emulates a projection where two classes overlap, while the second case presents good separation between classes. The cost associated with overlapping classes is higher. (**b**) 2D PCA projection and 2D t-SNE map of the MNIST handwritten digits dataset. With a significant overlap between classes seen in the PCA projection, the associated cost is higher than for the t-SNE map. (**c**) The $\hat{\mathcal {D}}(\sigma )$ curves corresponding to the 2D PCA projection and the 2D t-SNE map of the MNIST handwritten digits dataset.

### Detecting overlap between classes in categorical data

So far, we have shown examples where the cost was computed using continuous dependent variables. Here, we briefly explore the cost function application to categorical data. The dependent variable, $\phi$, can now be formed from numerical values of the class labels. Figure 8a shows in an illustrative way the capability of the $\hat{\mathcal {D}}(\sigma )$ metric to detect overlap between classes on a projection. The overlap between the two clouds of points, each representing one class, is reflected in an additional peak seen in the $\hat{\mathcal {D}}(\sigma )$ profile. When the clouds become sufficiently separated in space, $\hat{\mathcal {D}}(\sigma )$ exhibits a single rise. This behavior is then translated in the cost value, with the cost for the case with overlapping classes being greater. Next, we generate 2D projections of the MNIST handwritten digits dataset^74^ using PCA and t-SNE. The data observations are divided into ten classes, each representing one digit. We sample the full MNIST dataset by selecting only 1500 random samples from each class. The projections are visualized in Fig. 8b. The PCA projection introduces significant amount of overlap between classes and the cost associated with this projection is $\mathcal {L} = 4.4$. The cost for a much more unique t-SNE map, with clearly separated classes, is lower, $\mathcal {L} = 1.7$. Figure 8c additionally shows a comparison of the $\hat{\mathcal {D}}(\sigma )$ curves corresponding to the 2D PCA projection and the 2D t-SNE map, showing that the $\hat{\mathcal {D}}(\sigma )$ metric detected the significant class overlap in PCA. With some amount of scattered observations still present in the t-SNE map, the $\hat{\mathcal {D}}(\sigma )$ is not composed of a single rise as was seen in the fully non-overlapping classes example in Fig. 8a. This behavior in $\hat{\mathcal {D}}(\sigma )$ can be exploited to inform the appropriate hyper-parameter tuning to improve t-SNE maps. In the Supplementary material, we further demonstrate the potential of the cost function to guide the selection of an important hyper-parameter of t-SNE called perplexity.

## Discussion

Many factors can affect the quality of low-dimensional data parameterizations and there is a need to quantitatively assess those factors from the reduced-order modeling perspective. We propose a cost function that reduces the low-dimensional manifold topology to a single number. The two topological properties that the cost function pays attention to are uniqueness and feature sizes in relevant dependent variable(s). There are two main strengths of the proposed cost function. First, the manifold topology can be optimized for any target dimensionality. Second, the manifold topology can be optimized with respect to any user-selected dependent variables. Only the most important dependent variables need to be included in the manifold topology optimization. This can become particularly helpful in approaches where large state-spaces are compressed to a smaller number of parameters and regression is used to predict a physical quantity based on the compressed representation. Optimal manifolds can be found specifically from the perspective of the physical quantities of interest.

We demonstrate applications on numerically generated datasets and on experimental data containing noise. Our cost function can have useful applications in searching for the best data preprocessing strategies. This can include benchmarking existing strategies with newly invented ones. Often, those settings need to be tailored to a specific dataset and even to the target manifold dimensionality. In addition, various dimensionality reduction and manifold learning techniques can be assessed from the point of view of generating quality manifolds. A possible application might be to optimize the hyper-parameters to result in improved manifold topologies, especially for techniques such as t-SNE or UMAP which are known to strongly depend on hyper-parameter settings. While visual inspection of low-dimensional manifolds can be helpful, our cost function is a quantitative metric that can aid in qualitative assessments, especially when the manifold dimensionality exceeds 3D. Good quality of manifold topology is crucial when nonlinear regression is employed on manifolds. We show that improved projections can bring modeling benefits in regression tasks. We also briefly delineate possible applications of the cost function in dealing with categorical data where the cost function is computed from a dependent variable containing discrete values (class labels). For categorical data, the numerical values for the class labels and the distances between these values will affect the cost. Future work can investigate further the application to categorical data, especially for datasets where the number of classes becomes large or where classes exhibit a meaningful hierarchical structure, such as genomics or transcriptomics data. Future work can also include incorporating the cost function directly as an objective function in dimensionality reduction.

There remain some limitations of our proposed cost function. First, the computational cost for the normalized variance derivative, $\hat{\mathcal {D}}(\sigma )$, becomes high for large datasets. A potential solution to this restriction is to sample the dataset prior to computing the cost function. The Supplementary material sheds some light on how the cost function might respond to data sampling. Second, the current definition of the cost function cannot distinguish between the multiple scales of variation on a unique manifold and the non-uniqueness alone. This is due to the fact that the additional peaks in $\hat{\mathcal {D}}(\sigma )$ will show up in both scenarios, and both will increase the area under the $\hat{\mathcal {D}}(\sigma )$ curve. In our experience, however, the raise in $\hat{\mathcal {D}}(\sigma )$ due to non-uniqueness usually happens for $\sigma \ll \sigma _{peak}$, while the raise in $\hat{\mathcal {D}}(\sigma )$ due to varying feature sizes on an otherwise unique manifold happens at length scales closer $\sigma _{peak}$ (cf. Fig. 3d,f). The work in Ref.^30^ demonstrates how peak locations in $\hat{\mathcal {D}}(\sigma )$ due to non-uniqueness show a sensitivity to data sampling that peak locations due to unique features do not. Future work can consider incorporating information such as this into the cost function to better distinguish manifolds with non-uniqueness from those with small features. Finally, the resulting cost for a given manifold can only be interpreted in relation to cost(s) for other manifold(s). Thus, the cost function can help identify the best manifold among a set of manifolds, but, as of yet, no objective judgment can be made for a single cost value obtained for any one manifold. The same applies to cost associated with any one dependent variable on a single manifold—it should be interpreted in relation to costs for other dependent variables.

Nevertheless, we believe that this is a timely contribution that can help researchers across various disciplines and across numerous applications where low-dimensional data projections play an important role. Although we focused the demonstrations in this paper to four main datasets (argon plasma, reacting flows, atmospheric pollutant dispersion and categorical data), the cost function proposed can have broad applications in virtually any domain of science. We particularly look forward to exploring other applications, for instance on biological or medical data. We argue that further improvements in parameterization quality can be achieved in many areas of research if the low-dimensional parameter space is thoroughly explored and then assessed using the proposed cost function.

## Methods

### Data normalization

Prior to applying dimensionality reduction, datasets are often normalized. This can be especially beneficial if the dataset is composed of variables that have very different numerical ranges. Given a dataset composed of *Q* variables, $\mathbf {X} = [X_1, X_2, \dots , X_Q]$, we normalize each variable $X_j$ by subtracting its center, $c_j$, and dividing it by the scaling factor, $d_j$. In a matrix form, we can write the normalized dataset, $\tilde{\mathbf {X}}$, as:

1 $$\begin{aligned} \tilde{\mathbf {X}} = (\mathbf {X} - \mathbf {C}) \mathbf {D}^{-1}, \end{aligned}$$

where $\mathbf {C} \in {\mathbb {R}^{N \times Q}}$ is a matrix of centers with the *j*th column of that matrix populated with a value $c_j$ and $\mathbf {D} \in {\mathbb {R}^{Q \times Q}}$ is a diagonal matrix of scales with the *j*th element on the diagonal equal to $d_j$. In this work, we adopt several data scaling criteria collected in Table 1, where $s_j$ is the standard deviation and $k_j$ is the kurtosis of $X_j$. Techniques S1–S3 are newly introduced scaling techniques that we explore in this work. Throughout the main text, we refer to a particular scaling by its name as per Table 1. The name “None” is equivalent to no scaling applied to the dataset.

### Outlier removal

For the atmospheric pollutant dispersion dataset, we perform outlier detection and removal using the principal component classifier method^53,75^. Outliers are detected based on major and minor principal components (PCs). The observation *i* is classified as an outlier if the first PC classifier based on the *q* first (major) PCs:

2 $$\begin{aligned} \sum _{j=1}^q \frac{z_{ij}^2}{L_j} > c_1 \end{aligned}$$

or if the second PC classifier based on the $(Q-k+1)$ last (minor) PCs:

3 $$\begin{aligned} \sum _{j=k}^Q \frac{z_{ij}^2}{L_j} > c_2, \end{aligned}$$

where $z_{ij}$ is the (*i*, *j*)th element from the PCA-transformed data matrix and $L_j$ is the *j*th eigenvalue from PCA. Major PCs are selected such that the total variance explained is 50% (this determines the number *q*). Minor PCs are selected such that the remaining variance they explain is 20% (this determines the number *k*). PCA is performed with Auto scaling. Coefficients $c_1$ and $c_2$ are found such that they represent the quantile of the empirical distributions of the first and second PC classifier respectively. Here, we set the quantile to 98%, which allowed to find 509 outlying observations out of 18,540 total observations.

### Dimensionality reduction

A linear projection of a dataset onto a basis defined by $\mathbf {A} \in {\mathbb {R}^{Q \times q}}$ can be performed as:

4 $$\begin{aligned} \pmb {\eta } = \mathbf {X} \mathbf {A}, \end{aligned}$$

where $\pmb {\eta } = [\eta _1, \eta _2, \dots , \eta _q]$ defines the *q*-dimensional manifold parameters. In PCA, which is frequently used in this work, the basis matrix $\mathbf {A}$ is computed as the eigenvectors of a data covariance matrix. In the equation above, we assume that the dataset, $\mathbf {X}$, has already been appropriately preprocessed. The summary of linear and nonlinear dimensionality reduction techniques explored in this work is given in Table 2. For results reproducibility, random seed of 100 is used in all techniques that rely on randomness.

**Table 2:** Dimensionality reduction and manifold learning techniques used in this work. In the last column, we list the most important hyper-parameters of each method. The cost function can help fine-tune the hyper-parameters to achieve quality manifold topologies.

Technique	Type	Most important hyper-parameters
Principal component analysis (PCA)	Linear	None
Linear discriminant analysis (LDA)	Linear	None
Distance metric learning (DML)	Linear	Number of neighbors, distance metric
Multidimensional scaling (MDS)	Nonlinear	Distance metric
Locally linear embedding (LLE)^58^	Nonlinear	Number of neighbors
Hessian locally linear embedding (H-LLE)	Nonlinear	Number of neighbors
Local tangent space alignment (LTSA)	Nonlinear	Number of neighbors
Isomap^57^	Nonlinear	Distance metric
t-Distributed stochastic neighbor embedding (t-SNE)^55^	Nonlinear	Initialization, perplexity, learning rate, early exaggeration
Uniform manifold approximation and projection (UMAP)^60^	Nonlinear	Initialization, number of neighbors, distance metric
Spectral embedding (SE)	Nonlinear	Number of neighbors
Autoencoders (AE)^59^	Linear/nonlinear	Network architecture, activation function(s), learning rate

### PCA

We use the PCA implementation from the PCAfold Python package^77^ developed by the authors.

### LDA

We use the LDA implementation from the scikit-learn library. For the swiss roll dataset example, all parameters are set to default.

### DML

We use the the DML implementation from the pyDML Python package^76^. For the swiss roll dataset example, all parameters are set to default.

### MDS

We use the MDS implementation from the scikit-learn library. For the swiss roll dataset example, all parameters are set to default.

### LLE, H-LLE, LTSA

We use the LLE implementation from the scikit-learn library. For the swiss roll dataset example, all parameters are set to default, except the number of neighbors is set to 12. This applies to LLE, H-LLE and LTSA.

### Isomap

We use the Isomap implementation from the scikit-learn library. For the swiss roll dataset example, all parameters are set to default.

### t-SNE

We use the t-SNE implementation from the scikit-learn library. For the atmospheric pollutant dispersion dataset, all hyper-parameters are set to default, except the initialization which is set to PCA initialization. For the MNIST dataset and the swiss roll dataset example, all parameters are set to default.

### UMAP

We use the UMAP implementation from the umap-learn library^60^. For the atmospheric pollutant dispersion dataset, all hyper-parameters are set to default, except the number of neighbors is set to 12. For the swiss roll dataset example, all parameters are set to default.

### SE

We use the SE implementation from the scikit-learn library. For the swiss roll dataset example, all parameters are set to default, except the number of neighbors is set to 12.

### Autoencoder

We use the Keras Python library to set up the autoencoder for the swiss roll dataset example. For results reproducibility, random seed of 100 is used in TensorFlow (tf.random.set_seed(100)). The model architecture is 3-2-3 with hyperbolic tangent activations in all layers. The network weights are initialized from the Glorot uniform distribution and the biases are initially set to zeros. We use batch size of 50 and 200 epochs. The Adam optimizer is used with the learning rate 0.001 and the loss function is the mean squared error. We train the autoencoder model on 80% of the data.

### The proposed cost function

The starting point for formulating our cost function is computing the normalized variance proposed by Armstrong and Sutherland^30^. The goal is to assess the low-dimensional parameterization quality defined by *q* manifold parameters, $\pmb {\eta } \in {\mathbb {R}^{N \times q}}$, obtained using any dimensionality reduction or manifold learning technique. For length scales on a manifold given by the parameter $\sigma \in \langle \sigma _{min}, \sigma _{max} \rangle$, the normalized variance, $\mathcal {N}(\sigma )$, is computed for the *i*th dependent variable, $\phi _i$, as:

5 $$\begin{aligned} \mathcal {N}_i(\sigma ) = \frac{\sum _{k=1}^{N} (\phi _{k,i} - \mathcal {K}_i(\eta _k, \sigma ))^2 }{\sum _{k=1}^{N} (\phi _{k,i} - \bar{\phi _i})^2}, \end{aligned}$$

where *N* is the number of observations in a dataset, $\bar{\phi _i}$ is an arithmetic average of $\phi _i$, and $\mathcal {K}_i$ is computed as a weighted average of observations of $\phi _i$:

6 $$\begin{aligned} \mathcal {K}_i(\eta , \sigma ) = \frac{\sum _{j=1}^{N} w_j \phi _{j,i} }{\sum _{j=1}^{N} w_j }, \end{aligned}$$

where the weights, $w_j$, are determined using a Gaussian kernel:

7 $$\begin{aligned} w_j = \exp \Bigg ( \frac{- ||\eta _j - \eta ||_2^2}{\sigma ^2} \Bigg ). \end{aligned}$$

In Eq. (7), the quantity $||\eta _j - \eta ||_2^2$ is the squared Euclidean distance between the current location on a manifold, $\eta$, and any *j*th point on a manifold, $\eta _j$. We then construct the normalized variance derivative function as per^30^:

8 $$\begin{aligned} \mathcal {D}_i(\sigma ) = \frac{\mathrm {d} \mathcal {N}_i(\sigma )}{\mathrm {d} \log _{10} (\sigma )} + \lim _{\sigma \rightarrow 0} \mathcal {N}_i(\sigma ) \end{aligned}$$

and normalize it by its maximum value:

9 $$\begin{aligned} \hat{\mathcal {D}}_i(\sigma ) = \frac{\mathcal {D}_i(\sigma )}{\max (\mathcal {D}_i(\sigma ))}. \end{aligned}$$

The basis for the cost function proposed in this work is integration of the penalized normalized variance derivative function, $\hat{\mathcal {D}}(\sigma )$, over length scales given by $\sigma$. For the *i*th dependent variable, $\phi _i$, the area under the $\hat{\mathcal {D}}_i(\sigma )$ curve can be computed as:

10 $$\begin{aligned} A_i = \int _{\widetilde{\sigma }_{min, i}}^{\widetilde{\sigma }_{max, i}} \hat{\mathcal {D}}_i(\sigma ) \mathrm {d} \widetilde{\sigma }, \end{aligned}$$

where the tilde denotes a $\log _{10}$-transformed quantity (e.g. $\widetilde{\sigma } = \log _{10}\sigma$). We compute the area in the $\log _{10}$-space of the length scales $\sigma$ so that all scales of variation with different orders of magnitude are treated equally. We further introduce two penalties when computing the area:

1. Penalty for peak locations relative to the rightmost peak, $\sigma _{peak}$. This favors large feature sizes on a manifold over small ones.
2. Penalty for the area under $\hat{\mathcal {D}}(\sigma )$ happening at $\sigma < \sigma _{peak}$, and especially for $\sigma \ll \sigma _{peak}$. This penalizes multiple scales of variation that might show up as additional peaks in $\hat{\mathcal {D}}(\sigma )$ and becomes particularly useful when these additional peaks can be linked to non-uniqueness.

The cost function proposed herein takes these two penalties into account and is defined for $\phi _i$ as:

11 $$\begin{aligned} \mathcal {L}_i = \int _{\widetilde{\sigma }_{min, i}}^{\widetilde{\sigma }_{max, i}} P_i(\sigma , \sigma _{peak, i}) \cdot \hat{\mathcal {D}}_i(\sigma ) \mathrm {d} \widetilde{\sigma }, \end{aligned}$$

where $P_i(\sigma , \sigma _{peak, i})$ is the penalty function defined as:

12 $$\begin{aligned} P_i(\sigma , \sigma _{peak, i}) = \big | \widetilde{\sigma } - \widetilde{\sigma }_{peak, i} \big |^r + b \cdot \frac{\widetilde{\sigma }_{max, i} - \widetilde{\sigma }_{min, i}}{\widetilde{\sigma }_{peak, i} - \widetilde{\sigma }_{min, i}}. \end{aligned}$$

The first term in $P_i$ penalizes non-zero values in $\hat{\mathcal {D}}_i(\sigma )$ at length scales $\sigma < \sigma _{peak}$. It will especially amplify any area under the $\hat{\mathcal {D}}_i(\sigma )$ curve happening for $\sigma \ll \sigma _{peak}$. By increasing the power, the user can increase the amount of penalty for the variance occurring at $\sigma \ll \sigma _{peak}$. The second term in $P_i$ introduces a gentle penalty for the location of the rightmost peak and essentially increases or decreases the entire penalty function by a constant value. This second term acts to reward larger feature sizes as those should be easier to model. The parameter *b* is another hyper-parameter which controls the amount of the constant vertical shift of the entire penalty function. By increasing *b*, the user can increase the amount of penalty for the rightmost peak location, $\sigma _{peak}$. Throughout this work, we use $r=1$ and $b=1$. For completeness, in the next section, we illustrate the effect of setting *r* and *b* to values other than unity.

Python implementation of $\mathcal {N}(\sigma )$, $\hat{\mathcal {D}}(\sigma )$ and the proposed cost function, $\mathcal {L}$, has been developed by the authors and is available in the PCAfold software package^77^. In practice, numeric integration of $\hat{\mathcal {D}}(\sigma )$ is performed using a composite trapezoid rule. The peak values in $\hat{\mathcal {D}}(\sigma )$ are computed using the scipy.find_peaks function, which finds all local maxima by comparing the neighborhood of all discrete values of $\hat{\mathcal {D}}(\sigma )$. The value for $\sigma _{peak}$ is taken as the rightmost peak found by scipy.find_peaks, but the user can also select a percentage, *p*, of the value $\sigma _{peak}$ (such that the true rightmost peak becomes $p (\sigma _{max} - \sigma _{peak})$). Our recommended range for the parameter $\sigma$ is $\langle 10^{-7}; 10^3 \rangle$, with logarithmically-spaced in-between values. For instance, throughout this work we typically set sigma = numpy.logspace(-7, 3, 200). Throughout this work, the manifold parameters $\pmb {\eta }$ are scaled to a unit box (each $\eta _i$ is scaled to a [0, 1] range) before computing the cost. This is done so that the length scale, $\sigma$, has the same meaning in each manifold dimension.

### Effect of hyper-parameters *r* and *b* on the cost function

**Figure 9:**
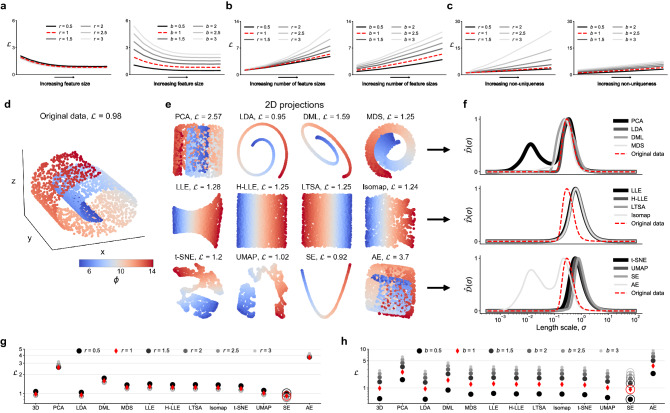
Illustrative demonstration of the effect of changing the cost function’s hyper-parameters, *r* and *b*. (**a**–**c**) Costs corresponding to toy functions from Fig. 3 when setting *r* and *b* to non-unity values. (**d**) The original 3D swiss roll dataset with the cost reported for the original 3D parameterization using $r = 1$ and $b = 1$. (**e**) 2D swiss roll dataset projections with the resulting costs using $r = 1$ and $b = 1$. (**f**) Comparison of the $\hat{\mathcal {D}}(\sigma )$ curves between generated 2D swiss roll dataset projections. The $\hat{\mathcal {D}}(\sigma )$ curve for the original 3D parameters is shown with the red dashed line for reference. (**g**) Costs for the 3D swiss roll data parameters and its various 2D projections with *b* set fixed to unity, and with changing *r*. (**h**) Costs for the 3D swiss roll data parameters and its various 2D projections with *r* set fixed to unity, and with changing *b*. With the circular outlines in (**g**,**h**), we mark the lowest costs for any of the 2D projections which consistently happens for the SE manifold, irrespective of the values selected for *r* and *b*.

The hyper-parameters *r* and *b* from Eq. (12) may be changed to emphasize penalties applied to non-uniqueness and/or small feature sizes when computing overall costs. Figure 9 illustrates the effect of setting *r* and *b* to non-unity values. In Fig. 9a–c, we utilize the toy functions from Fig. 3 and show how $\mathcal {L}$ behaves for various *r* and *b*. For clearer analysis, whenever *r* is varied, *b* is set to unity, and vice versa. For comparison, with the red dashed lines, we mark costs corresponding to $r=1$ and $b=1$. In principle, increasing *b* increases $\mathcal {L}$ more severely for manifolds where a dependent variable exhibits small feature sizes (see Fig. 9a). Conversely, increasing *r* for unique manifolds does not affect the $\mathcal {L}$ values significantly. As can be understood from Fig. 9c, increasing *r* increases $\mathcal {L}$ more severely for manifolds with non-uniqueness. Even if non-uniqueness is present on a manifold with a fixed largest feature size, changing *b* does not affect the $\mathcal {L}$ values significantly. For a unique manifold with multiple feature sizes, increasing *r* or *b* can increase $\mathcal {L}$ by a similar amount (Fig. 9b). Due to the blending of multiple scales of variation in the $\hat{\mathcal {D}}(\sigma )$ profile seen in Fig. 3d, we have also applied a $p=70\%$ shift in $\sigma _{peak}$ in Fig. 9b, as per discussion in the previous section. In Fig. 9a–c, the overall trend of the cost function is preserved when changing *r* and *b*.

We further test the impact of *r* and *b* on the verdict given by the cost function across various dimensionality reduction and manifold learning techniques. We use the classic 3D swiss roll dataset and generate various 2D projections. The original 3D topology of this dataset can be seen in Fig. 9d, colored by a dependent variable, $\phi$. The cost corresponding to the parameterization in the original 3D space is $\mathcal {L} = 0.98$ with $r=1$ and $b=1$. Figure 9e shows twelve different 2D projections of that dataset generated with various linear and nonlinear techniques. The cost value, $\mathcal {L}$, is reported for each projection taking $r=1$ and $b=1$. Among all the reduction techniques selected for this demonstration, PCA and a linear autoencoder (AE) introduce the most significant non-uniqueness. The costs for AE and PCA projections are the highest, while SE, LDA and UMAP show the lowest costs. The small cost for SE, LDA or UMAP projections (comparable to the cost in the original 3D space) is likely due to a combination of two factors: projection uniqueness and large feature sizes created through sufficient separation of distinct $\phi$ values in space. Figure 9f shows the $\hat{\mathcal {D}}(\sigma )$ curves for the original 3D data (red dashed lines) and for the 2D projections, in groups of four. The location of $\sigma _{peak}$ is shifted to the right for the SE and UMAP projections with respect to the original 3D data parameters. A similar observation holds for LLE, H-LLE, LTSA and Isomap projections. This can be due to a relatively large distance between the smallest and the largest $\phi$ values on these projections. In the original 3D space, the high and low values of $\phi$ are relatively close to each other due to manifold curvature. In Fig. 9g,h, we show the effect on the cost function evaluations of the various swiss roll data projections when changing the hyper-parameters *r* and *b*. In Fig. 9g, costs increase with increasing *r* only for the PCA and AE projections. This is understandable, since those are the only projections with significant levels of non-uniqueness. Increasing the hyper-parameter *r* thus increasingly amplifies the “secondary” rise seen in the $\hat{\mathcal {D}}(\sigma )$ profile at $\sigma < \sigma _{peak}$ for PCA and AE. Interestingly, this behavior with *r* can potentially be used to detect manifolds with non-uniqueness among a set of parameterizations. In Fig. 9h, increasing *b* increases costs for all parameterizations (3D and 2D). Costs increase more rapidly for PCA and AE projections than for any other explored projection (note the logarithmic scale of the vertical axis). This is also due to emphasizing the “secondary” rise seen in the $\hat{\mathcal {D}}(\sigma )$ profile when computing the penalized area, but this time through increasing the vertical shift of the entire penalty function. With the circled outlines in Fig. 9g,h, we mark the lowest cost that happened for any of the 2D projections across the explored values of *r* and *b*. Among the *r* and *b* values explored, the smallest cost consistently happens for the SE projection. We also note that the ratio between $\mathcal {L}$ for the worst projection (AE) and $\mathcal {L}$ for the best projection (SE) is generally higher when *r* is set above unity than when *b* is set above unity. Thus, increasing *r* above unity can help create clearer separations in $\mathcal {L}$ values when ranking manifolds with varying levels of non-uniqueness.

### On the computational impact of evaluating the cost function

We note that the computational time required to compute $\mathcal {L}$ for a dataset with *N* observations and for *m* dependent variables scales as $\mathcal {O}(m N^2)$, assuming the same number of discrete values of $\sigma$. The code for computing the cost function, that we provide in the PCAfold library, is parallelized with respect to $\sigma$, since the normalized variance computations are entirely independent of one another for different values of $\sigma$. Thus, our code can be readily ran on multiple CPUs, which we generally recommend for $N>10^5$. In the Supplementary material, we show the effect of data sampling on the cost function. We note, that while subsampling the data can ease the computational cost of $\mathcal {L}$, it has an enhanced effect on dependent variables which exhibit variation at multiple scales (e.g. from non-uniqueness). A possible future implementation that could reduce the computational time of evaluating Eq. (6) is to approximate the weighted average with a sum over points within/near the currently considered length scale, $\sigma$. This implementation can make the computational time scale approximately as $\mathcal {O}(m N)$ for small $\sigma$ and only scale as $\mathcal {O}(m N^2)$ as $\sigma$ encompasses all points on a manifold.

There is a second aspect to the question of the computational cost, that is more elusive to quantify, and that is the time required for the researcher to find a good manifold topology through trial and error exploration, if no quantitative tools were applied to guide the choice of a manifold. We argue that this second aspect can become a bottleneck in effective application of reduced-order models. In this regard, one may find that our cost function allows for “quick” assessments of projections resulting from a range of dimensionality reduction and manifold learning techniques, as well as a variety of data preprocessing strategies applied to the training data. Moreover, since $\mathcal {L}$ can be implemented in optimization tasks, it is likely that an optimum in manifold topologies is found in an automated way, compared to a trial and error approach.

### $\mathcal {L}$-informed feature selection

We developed a feature selection algorithm that iteratively eliminates state variables from the dataset based on minimizing the cost, $\mathcal {L}$, of PCA projections^65^. There are three inputs to the algorithm: the original dataset, $\mathbf {X} \in {\mathbb {R}^{N \times Q}}$, the target dependent variables, $\pmb {\phi } \in {\mathbb {R}^{N \times m}}$, and the target manifold dimensionality, *q*. The pseudocode below shows in closer detail how the algorithm computes the optimized subset of the original state variables. At each iteration *i*, the algorithm computes PCA projections resulting from removing each variable, one at a time. At the end of the iteration, the variable whose removal decreased the cost value the most is discarded from the dataset. At the next iteration, the process repeats, but now on a dataset with one less variable. We only allow $Q-q$ iterations so that we never reduce original data dimensionality below *q* requested by the user. Once all iterations have finished, the algorithm looks back at final costs from all iterations and returns the optimized subset, $\mathbf {X}_S$, corresponding to the iteration that showed the minimum cost value. Such subset should then generate an optimized manifold topology. The algorithm is available in the PCAfold software package^77^.
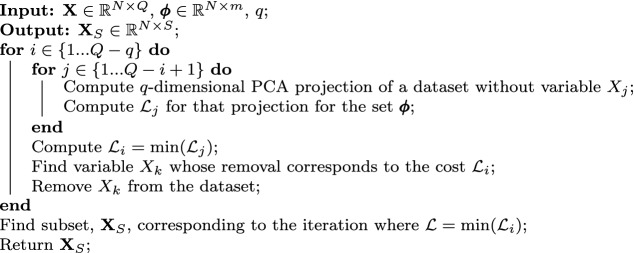


### Nonlinear regression using artificial neural networks (ANNs)

In the atmospheric pollutant dispersion example, we use nonlinear regression using ANNs. We use the Keras Python library to set up the ANN regression. For results reproducibility, random seed of 100 is used in TensorFlow (tf.random.set_seed(100)). The inputs of the network are the three manifold parameters, $\pmb {\eta } = [\eta _1, \eta _2, \eta _3]$, either from PCA, UMAP, Isomap or t-SNE. The output is the turbulent Schmidt number, $\phi = Sc_t$. The model architecture is 2-5-5-1 for 2D projections and 3-5-5-1 for 3D projections with sigmoid activations in all layers except the output layer, where we use linear activation. The network weights are initialized from the Glorot uniform distribution and the biases are initially set to zeros. We use batch size of 100, validation split of 0.2 and 500 epochs. The Adam optimizer is used with the learning rate 0.001 and the loss function is the mean squared error. We train the regression model on 80% of the data and measure the MAE on the remaining 20% test data not seen by the ANN model. The results reported in Fig. 7 are for the 20% test data.

### Nonlinear regression using kernel regression

In the atmospheric pollutant dispersion example, we use kernel regression from the PCAfold software package^77^. We use the Nadaraya-Watson estimator with a Gaussian kernel with a varying bandwidth. The bandwidth is determined locally based on 50 nearest neighbors of the query point. We train the regression model on 80% of the data and measure the MAE on the remaining 20% test data not seen by the kernel regression model. The results reported in Fig. 7 are for the 20% test data.

### Reacting flow data generation

The reacting flow datasets for combustion of syngas in air and hydrogen in air were generated using Spitfire Python package^78^ available at: github.com/sandialabs/Spitfire. The datasets were generated using a steady laminar flamelet model for a range of dissipation rates from chemical equilibrium to extinction and a range of mixture fractions between 0 and 1. For the syngas/air dataset, the fuel stream is composed of a mixture of carbon monoxide and hydrogen in 10:1 molar proportion^79^. The oxidizer stream is air. Both streams have initial temperature 300 K and pressure 101,325 Pa. For the hydrogen/air dataset, the fuel stream is composed of hydrogen and the oxidizer stream is air^80^. Both streams have initial temperature 300 K and pressure 101,325 Pa. For both fuels, the mixture fraction grid is denser closer to the stoichiometric conditions.

## Supplementary Information

Supplementary Information.

## Data Availability

The datasets generated during or analyzed during the current study are available in the GitHub repository: https://github.com/kamilazdybal/cost-function-manifold-assessment. The atmospheric pollutant dispersion dataset^46^ and the argon plasma dataset^66,67^ are property of Université libre de Bruxelles. The Sandia flames data^69^ can be accessed at: tnfworkshop.org/data-archives/pilotedjet/ch4-air.

## Code availability

The code used during the current study is publicly available in the GitHub repository: https://github.com/kamilazdybal/cost-function-manifold-assessment.
