# Supplementary material for: Cost function for low-dimensional manifold topology assessment
Source: Sci Rep. 2022 Aug 25;12:14496. doi: 10.1038/s41598-022-18655-1 (PMC9411209; doi:10.1038/s41598-022-18655-1)
Supplement: Supplementary file 1 — Supplementary Information. [file 41598_2022_18655_MOESM1_ESM.pdf]

Supplementary material for:
Cost function for low-dimensional manifold topology assessment

Kamila Zdybał[1, 2, *], Elizabeth Armstrong[3], James C. Sutherland[3], and Alessandro Parente[1, 2]

[1]*Université Libre de Bruxelles, École polytechnique de Bruxelles, Aero-Thermo-Mechanics Laboratory, Brussels, Belgium*
[2]*Université Libre de Bruxelles and Vrije Universiteit Brussel, Combustion and Robust Optimization Group (BURN), Brussels, Belgium*
[3]*Department of Chemical Engineering, University of Utah, Salt Lake City, Utah, USA*
[*]*kamila.zdybal@ulb.ac.be*

Available code

All code used to produce the results in the original publication and in this supplementary material can be found in the form of Jupyter notebooks in the public GitHub repository:
</> `github.com/kamilazdybal/cost-function-manifold-assessment`

Contents

S1 Tuning t-SNE hyper-parameters 1

S2 Extended results for the relation between $\mathcal{L}$ and the nonlinear regression performance 5

S3 Sensitivity to data sampling 7

S1 Tuning t-SNE hyper-parameters

Perplexity is an important hyper-parameter of t-SNE. It describes the effective number of data points that are considered as neighbors. The topology of the low-dimensional t-SNE projection can be highly sensitive to the choice of perplexity [1]. Generally, small perplexity allows to uncover fine structures in the data, while high perplexity maintains the global structure of the data. Values recommended in the original t-SNE publication are between 5 and 50 [2], with a default value set to 30 in the scikit-learn implementation [3]. Recently, a study done by Kobak and Berens [4] shed more light on how to set the perplexity for transcriptomics data. As a result, they proposed to use perplexity equal to $n/100$, where n is the number of observations in the data. However, it is not clear whether such rules will carry over to other types of data, or if there exists a yet better setting. Other important parameters of t-SNE include: the learning rate which affects the gradient descent optimization, early exaggeration which determines how strongly neighboring data points should be attracted to each other during the first few iterations, or type of initialization. In this section, we demonstrate the potential of the proposed cost function to optimize t-SNE hyper-parameters. We focus on the perplexity parameter.

The influence of the perplexity parameter on the quality of the resulting t-SNE projection is illustratively demonstrated in Fig. S1. We create a synthetic 3D dataset visualized in Fig. S1a and we create an artificial dependent variable, ϕ, from a bivariate Gaussian function. In Figs. S1b-c, we apply t-SNE to the synthetic dataset using perplexity=1 and perplexity=30 respectively. The cost associated with the projection corresponding to perplexity=1 is $\mathcal{L} = 1.3$ and corresponding to perplexity=30 is $\mathcal{L} = 0.9$. The higher cost can be understood by looking at the projection colored by ϕ, where the very fine thread-like structures grouped tightly together introduce increased variation

in the dependent variable values at small length scales. The smaller cost associated with perplexity=30 is a result of a much smoother change in the dependent variable values over the same manifold length scales. In Fig. S1d, a comparison of the $\hat{\mathcal{D}}(\sigma)$ curves for the two t-SNE maps corresponding to two perplexity settings confirms that setting perplexity=1 introduced more variance in ϕ at smaller manifold length scales than setting perplexity=30. For comparison, with the red dashed line we also show the $\hat{\mathcal{D}}(\sigma)$ curve for the dataset in the original 3D space.

We now explore how the cost function ranks t-SNE projections when perplexity is changed discretely from 1 to 16 for another synthetic dataset. This time, the dataset is created as a uniform 2D grid and an artificial dependent variable is again created from a bivariate Gaussian function. This synthetic dataset has 400 observations. Fig. S2a visualizes the resulting 2D t-SNE projections for each perplexity value, along with the costs, $\mathcal{L}$. The highest cost corresponds to perplexity=4, where the 2D projection fragments the data observations into smaller groups. Starting from perplexity=6, the costs remain at a relatively same level, but for perplexity=15, we see another slight increase in the cost value. The corresponding t-SNE projection has one corner of the 2D square grid turned "inside out", which changes the relative feature sizes on the projection. The smallest cost value happens for the perplexity=7 case. Fig. S2b visualizes in closer detail the $\hat{\mathcal{D}}(\sigma)$ curves corresponding to the perplexity=7 case, and to the perplexity=15 case. We have selected the perplexity=15 case to observe the effect of the visible "corner flipping" on the $\hat{\mathcal{D}}(\sigma)$ curve, as compared to the $\hat{\mathcal{D}}(\sigma)$ curve corresponding to the smallest cost value. With the corner of the grid turned "inside out", the relative change in the largest feature sizes on a manifold is distorted and is reflected in shifting the σ_{peak} location to the left with respect to the perplexity=7 case.

As discussed in the Methods section in the main text, before computing the cost function, we always scale each manifold parameter, η_i, to a $[0, 1]$ range, which we refer to as a "unit box" scaling (we note here that this is different from scaling the training dataset). One more interesting observation that can be drawn from Fig. S2 is that when a projection is scaled to a unit box before computing the cost function, the rotation of a manifold can affect the cost value. The reason for this is that by first rotating the projection, and then scaling it to a unit box, we effectively compress the global feature sizes on a projection. Thus, at the same length scale, σ, we can detect more variance when feature sizes are compressed. The lowest $\mathcal{L}$ in Fig. S2 is observed for perplexity=7, where the rotation of a projection is such that the square structure of the projection aligns with the vertical and horizontal axis. Thus, the edge length of the square structure of the projection is close to 1. In comparison, for perplexity set to a value between 8 and 14, the rotation of the projection is such that the edge length of the square grid structure becomes smaller than 1. With the same dependent variable coloring the projections, the same variance is occurring at smaller length scales on projections corresponding to perplexity between 8 and 14 than corresponding to perplexity=7. Nevertheless, manifold rotation is expected to have a benign effect on $\mathcal{L}$, as compared to non-uniqueness or varying feature sizes.

We note that the verdict given by $\mathcal{L}$ on the best projection is entirely related to the dependent variable assessed on a projection. The potential strength of our proposed quantitative cost function is that even when no relevant dependent variable exists *a priori* for a given manifold, an artificial dependent variable can be created by the user, as we have done in this example.

Finally, we perform a brief exploration of setting the perplexity parameter to a value dependent on the total number of observations in a dataset, as per the methodology proposed by Kobak and Berens [4]. We use the categorical MNIST dataset with varying total number of observations, n. We create varying n by subsampling the MNIST dataset and taking 500, 1000, 1500, 2000, 3000, 4000, 5000 and 6000 samples from each of the ten classes. For each n, we set the perplexity to: $n/1000$, $n/500$, $n/250$, $n/200$, $n/150$, $n/125$, $n/100$, $n/90$, $n/80$ and $n/70$. We then measure the cost of the resulting 2D t-SNE projection. Fig. S3 shows the costs, $\mathcal{L}$, for the class labels as the only dependent variable (similarly as we have done in Fig. 8 in the main text). The value $n/100$ is the one proposed in [4] for categorical transcriptomics datasets and is marked with the red circle in Fig. S3. The perplexity value corresponding to the smallest $\mathcal{L}$ is marked with a shaded outline for each n. There is a generally decreasing trend in the cost function with increasing the perplexity value. This suggests that settings such as $n/1000$ or $n/500$ are too small for the MNIST dataset. We note that while the setting $n/100$ never leads to the smallest $\mathcal{L}$ in Fig. S3, the value $n/100$ lies in the region where the costs plateau. This suggests that $n/100$ can be a good approximation for the perplexity value. We note that the results reported in Fig. S3 are tied to the MNIST dataset, and might not generalize to other categorical datasets. Similar test as we have done here can be performed for continuous dependent variables. Future work can include testing the perplexity setting on other categorical datasets, such as the transcriptomics data used in [4]. We note that more comprehensive tests should be performed to satisfactorily address the question of setting the perplexity parameter as dependent on the number of data observations. Since the cost function takes information about the relevant dependent variable(s), the choice of the dependent variable can potentially affect the verdict for the perplexity setting. In addition, several questions still need to be addressed in

future work when applying the cost function to categorical data. We have briefly pointed those out in the Discussion section in the main text.

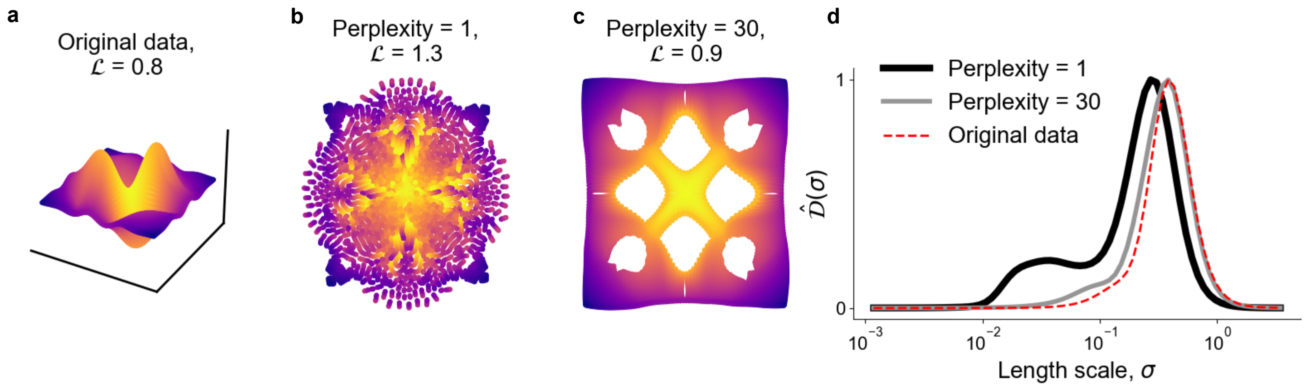

Figure S1: Topologies of t-SNE projections can be very different depending on the perplexity parameter. a A synthetic 3D dataset and its 2D t-SNE projections with **b** perplexity=1, and **c** perplexity=30. It can be seen that small perplexity uncovers fine structures in the data, while a higher perplexity maintains the global structure of the data. The cost corresponding to the manifold obtained using perplexity=1 is higher in comparison. **d** Comparison of the $\hat{\mathcal{D}}(\sigma)$ curves for the t-SNE maps corresponding to two perplexity settings. The $\hat{\mathcal{D}}(\sigma)$ curve for the original 3D parameters is shown with the red dashed line for reference.

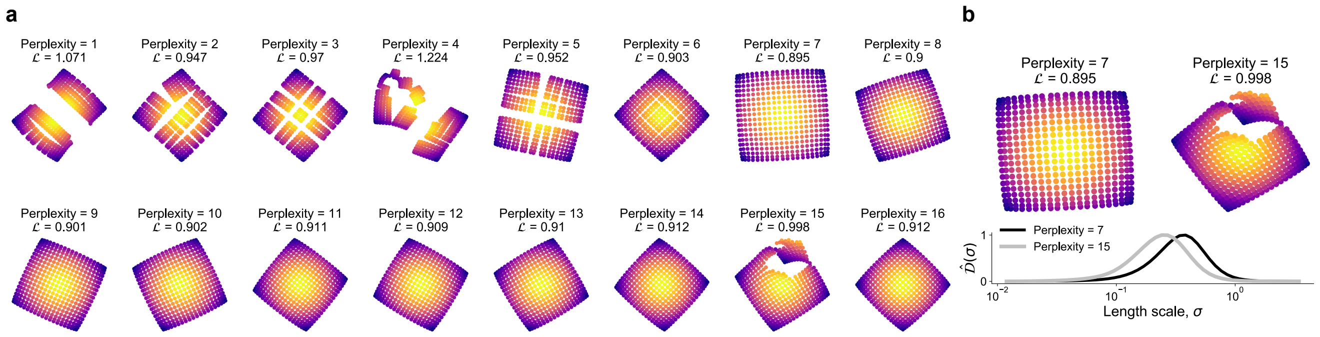

Figure S2: Ranking of 2D t-SNE projections of a synthetic dataset while varying the perplexity parameter. a 2D t-SNE projections when perplexity is varied from 1 to 16 with the cost value, $\mathcal{L}$, reported for each projection. **b** Comparison of costs, $\mathcal{L}$, and $\hat{\mathcal{D}}(\sigma)$ curves for two example t-SNE projections corresponding to perplexity=7 and perplexity=15.

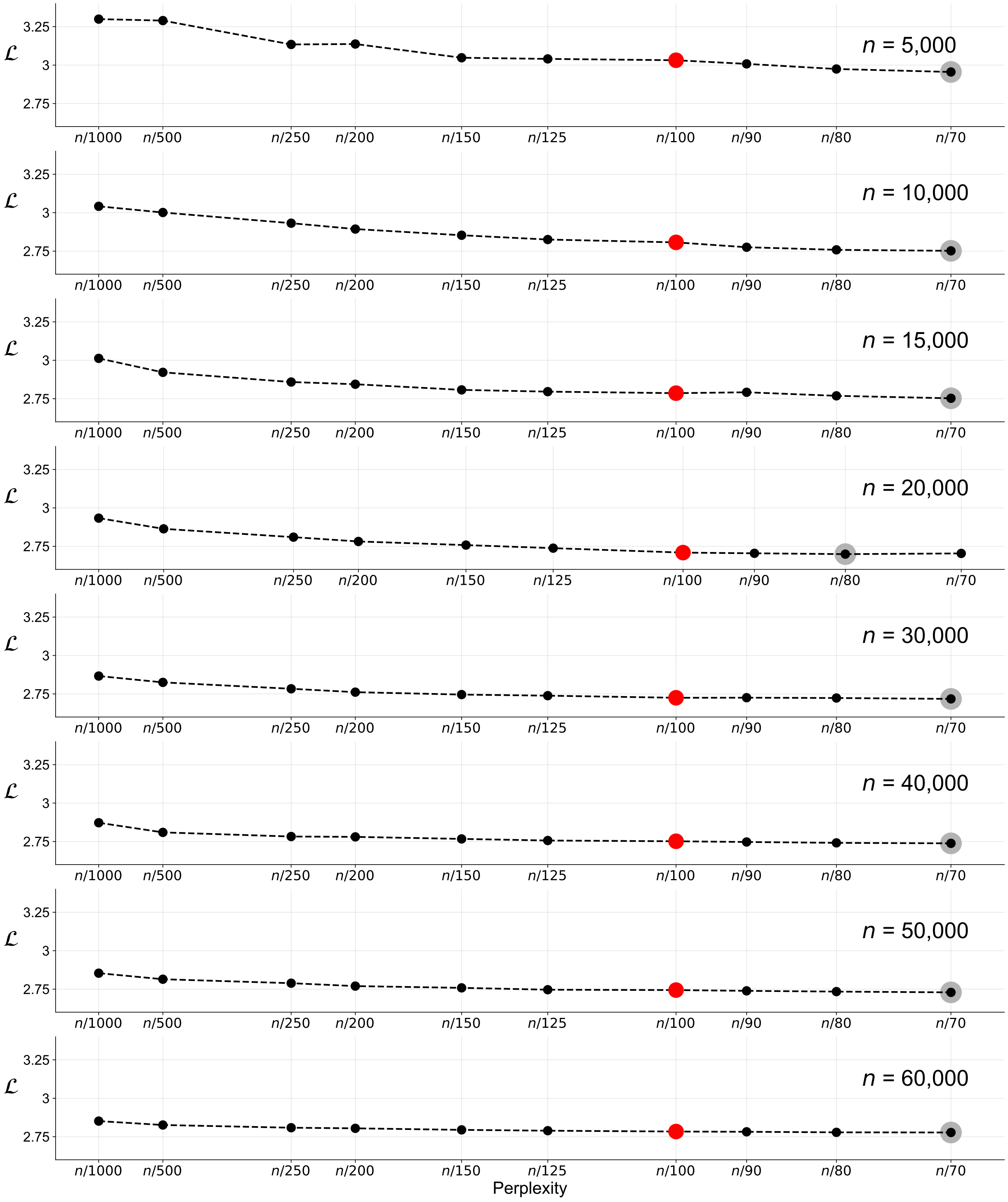

Figure S3: Setting the perplexity parameter to a value dependent on the total number of observations, n, in a dataset. We show costs, $\mathcal{L}$, obtained for 2D t-SNE projections of the MNIST dataset with varying number of total observations, n. The dependent variable are the class labels. n is varied from 5,000 to 60,000 through selecting between 500 to 6,000 samples from each class. We test ten different perplexity settings, ranging from $n/1000$ to $n/70$. With the red circle we mark the perplexity setting $n/100$, proposed by Kobak and Berens [4]. With the shaded outline, we mark the smallest cost that happened for any of the ten perplexity settings.

S2 Extended results for the relation between $\mathcal{L}$ and the nonlinear regression performance

Fig. S4 is analogous to Fig. 7 presented in the main text, but the cost function is measured based on 3D PCA and t-SNE projections. Nonlinear regression using ANN and kernel regression is thus performed based on three independent manifold parameters. We report Spearman correlation coefficient between $\mathcal{L}$ and MAE. The same observation as was made for 2D projections applies here, that correlations are higher for ANN regression than for kernel regression.

Figs. S5-S6 contain the same results as Fig. 7 from the main text and Fig. S4 respectively, but show each scaling technique on a separate scatter plot. Figs. S5-S6 thus allow to better observe the non-negligible impact that data normalization has on the manifold topology, and consequently, on the nonlinear regression performance.

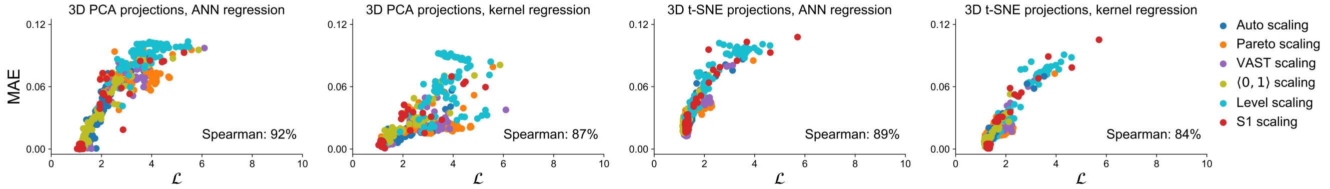

Figure S4: Relating the cost function to nonlinear regression predictions based on various low-dimensional projections of the atmospheric pollutant dispersion data. Scatter plots of $\mathcal{L}$ versus MAE from ANN and kernel regression predictions of Sc_t. We show predictions based on 600 different 3D PCA projections, and based on 600 different 3D t-SNE projections as the independent manifold parameters. We test a few selected scaling techniques applied to the atmospheric dispersion data; legend applies to all figures.

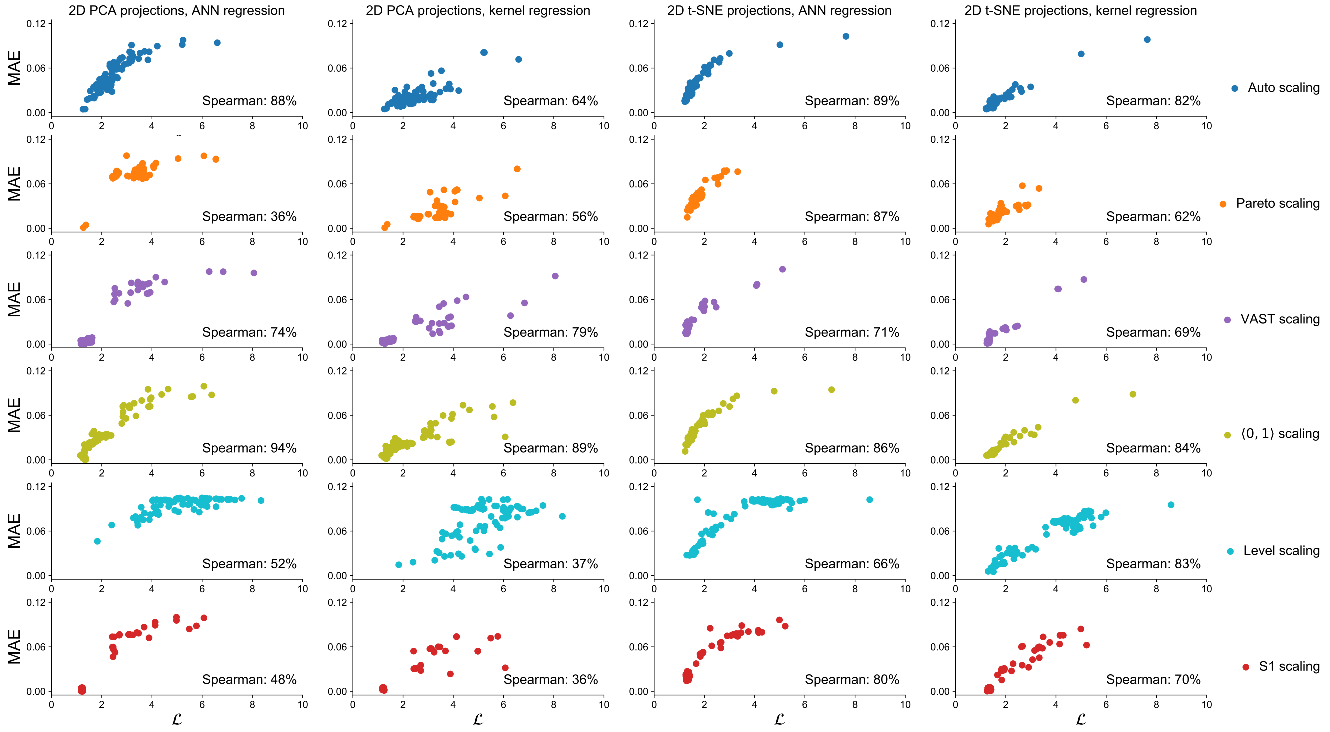

Figure S5: Relating the cost function to nonlinear regression predictions based on various low-dimensional projections of the atmospheric pollutant dispersion data. Scatter plots of $\mathcal{L}$ versus MAE from ANN and kernel regression predictions of Sc_t. This figure contains the same results as Fig. 7 in the main text, but we present each scaling technique separately. Each scatter plot contains 100 data points.

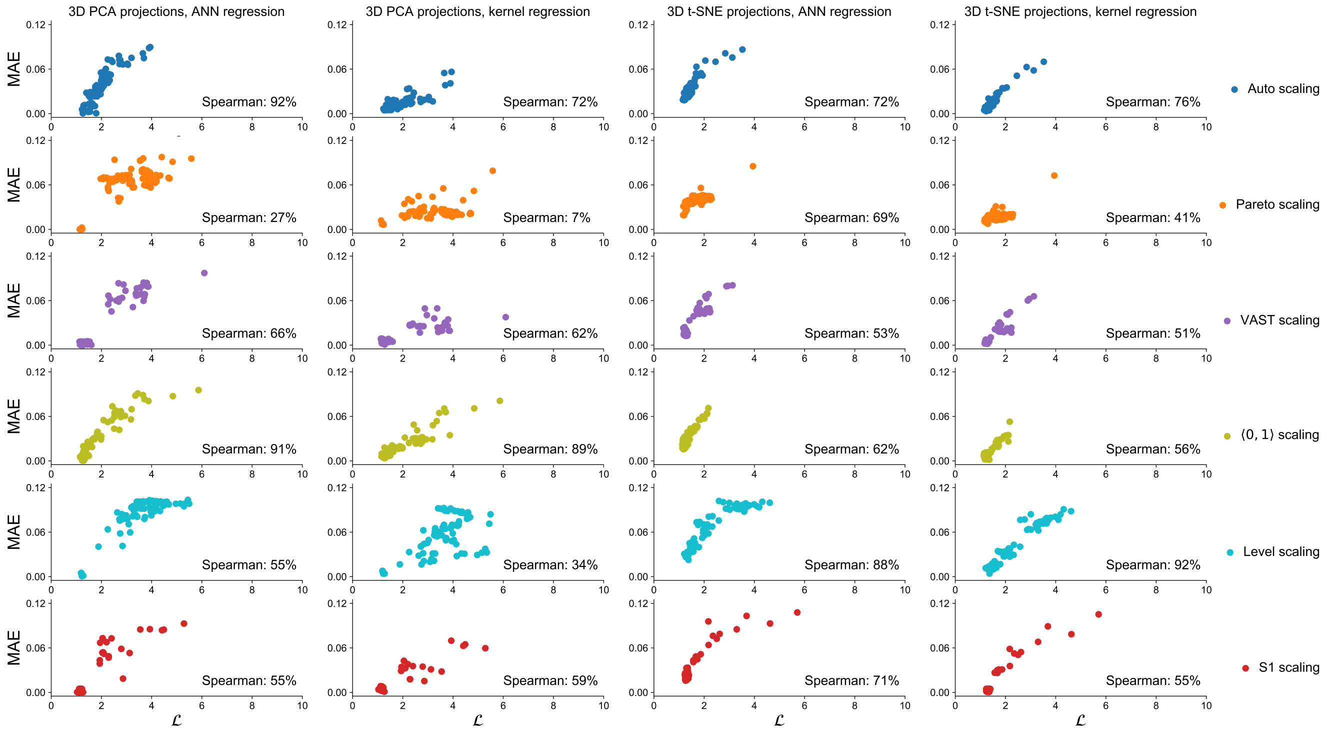

Figure S6: Relating the cost function to nonlinear regression predictions based on various low-dimensional projections of the atmospheric pollutant dispersion data. Scatter plots of $\mathcal{L}$ versus MAE from ANN and kernel regression predictions of Sc_t. This figure contains the same results as Fig. S4, but we present each scaling technique separately. Each scatter plot contains 100 data points.

S3 Sensitivity to data sampling

With the high computational cost for computing the normalized variance derivative, $\hat{\mathcal{D}}(\sigma)$, on large datasets, we pointed the reader towards data sampling as a possible mitigation strategy. In this section, we shed some light on how the cumulative cost value, $\mathcal{L}$, changes when the observations on a manifold are randomly sampled at 5%, 20%, 50%, and 80%. We compare these costs against taking a full set of observations (100%). Fig. S7 visualizes this change in cost value across various data scaling techniques and across datasets considered in the main text: reacting flow (originally 14,550 observations), argon plasma (originally 201,400 observations) and atmospheric pollutant dispersion data (originally 18,540 observations). For each dataset, we compute costs for 2D and 3D PCA projections. Since the argon plasma dataset is large, sampling was performed at 5%, 20%, 50% only, where 50% matches the sampling taken in the main text for that dataset. For the reacting flow dataset and the atmospheric physics dataset, we observe subtle changes in $\mathcal{L}$ when at least 50% of the data is taken. Generally, the largest change in the cost value happens when subsampling the data at 5% or at 20%, and usually when the cost for the entire dataset is high. This suggests that non-uniqueness might be more strongly affected by random data sampling and is in line with the reasoning presented in [5]. It is important to note however, that these results might not generalize to any dataset. They are merely meant to shed some light on how severely the cost function might respond to random sampling of observations from the manifold.

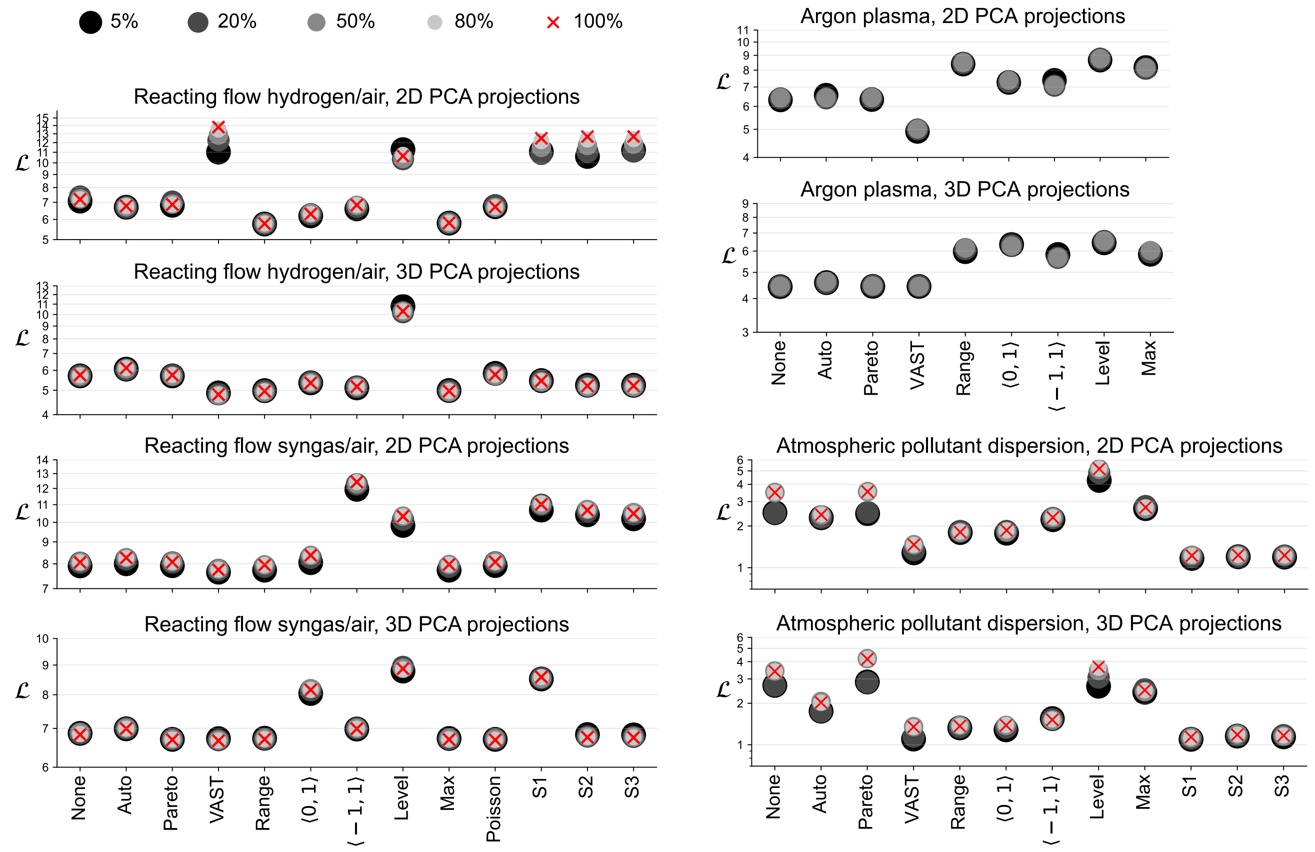

Figure S7: Sensitivity of the cost function to random sampling of the various datasets used in the main text. The costs are computed for 2D and 3D PCA projections and for various data scaling options. The same legend applies to all figures, where the red crosses mark costs corresponding to taking the full dataset and the percentage values represent 5%, 10%, 50% or 80% random samples from the full data.

References

[1] M. Wattenberg, F. Viégas, and I. Johnson. How to use t-SNE effectively. *Distill*, 2016.

[2] L. Van der Maaten and G. Hinton. Visualizing data using t-SNE. *Journal of Machine Learning Research*, 9(11), 2008.

[3] F. Pedregosa, G. Varoquaux, A. Gramfort, V. Michel, B. Thirion, O. Grisel, M. Blondel, P. Prettenhofer, R. Weiss, V. Dubourg, J. Vanderplas, A. Passos, D. Cournapeau, M. Brucher, M. Perrot, and E. Duchesnay. Scikit-learn: Machine learning in Python. *Journal of Machine Learning Research*, 12:2825–2830, 2011.

[4] D. Kobak and P. Berens. The art of using t-SNE for single-cell transcriptomics. *Nature communications*, 10(1):1–14, 2019.

[5] E. Armstrong and J.C. Sutherland. A technique for characterising feature size and quality of manifolds. *Combustion Theory and Modelling*, 0(0):1–23, 2021.
